# Are patients with cancer at higher risk of COVID-19-related death? A systematic review and critical appraisal of the early evidence

**DOI:** 10.1016/j.jcpo.2022.100340

**Published:** 2022-09

**Authors:** Victoria Freeman, Suzanne Hughes, Chelsea Carle, Denise Campbell, Sam Egger, Harriet Hui, Sarsha Yap, Silvia Deandrea, Michael Caruana, Tonia C. Onyeka, Maarten J. IJzerman, Ophira Ginsburg, Freddie Bray, Richard Sullivan, Ajay Aggarwal, Stuart J. Peacock, Kelvin K.W. Chan, Timothy P. Hanna, Isabelle Soerjomataram, Dianne L. O'Connell, Julia Steinberg, Karen Canfell

**Affiliations:** a The Daffodil Centre, The University of Sydney, a joint venture with Cancer Council NSW, Australia; b Directorate General for Health, Lombardy Region, Milano, Italy; c Environmental Health Unit, Agency for Health Protection, Pavia, Italy; d Department of Anaesthesia/Pain & Palliative Care Unit, Multidisciplinary Oncology Centre, College of Medicine, University of Nigeria, Nigeria; e University of Melbourne, Centre for Cancer Research and Centre for Health Policy, Australia; f Department of Cancer Research, Peter MacCallum Cancer Centre, Parkville, VIC, Australia; g Perlmutter Cancer Center and the Department of Population Health, NYU Grossman School of Medicine, New York, United States; h Cancer Surveillance Branch, International Agency for Research on Cancer, Lyon, France; i King's Institute Cancer Policy, King's College London, United Kingdom; j Department of Oncology, Guy's and St Thomas' NHS Foundation Trust, London, United Kingdom; k Department of Health Services Research and Policy, London School of Hygiene and Tropical Medicine, London, United Kingdom; l Canadian Centre for Applied Research in Cancer Control (ARCC), Canada; m Cancer Control Research, BC Cancer, Canada; n Faculty of Health Sciences, Simon Fraser University, Canada; o Sunnybrook Odette Cancer Centre, University of Toronto, Toronto, Canada; p Division of Cancer Care and Epidemiology, Cancer Research Institute at Queen’s University, Kingston, ON, Canada; q Department of Oncology and Department of Public Health Sciences, Queen’s University, Kingston, ON, Canada

**Keywords:** Neoplasms, COVID-19, SARS-CoV-2, Mortality

## Abstract

**Background:**

Early reports suggested that COVID-19 patients with cancer were at higher risk of COVID-19-related death. We conducted a systematic review with risk of bias assessment and synthesis of the early evidence on the risk of COVID-19-related death for COVID-19 patients with and without cancer.

****Methods and findings**:**

We searched Medline/Embase/BioRxiv/MedRxiv/SSRN databases to 1 July 2020. We included cohort or case-control studies published in English that reported on the risk of dying after developing COVID-19 for people with a pre-existing diagnosis of any cancer, lung cancer, or haematological cancers. We assessed risk of bias using tools adapted from the Newcastle-Ottawa Scale. We used the generic inverse-variance random-effects method for meta-analysis. Pooled odds ratios (ORs) and hazard ratios (HRs) were calculated separately.

Of 96 included studies, 54 had sufficient non-overlapping data to be included in meta-analyses (>500,000 people with COVID-19, >8000 with cancer; 52 studies of any cancer, three of lung and six of haematological cancers). All studies had high risk of bias. Accounting for at least age consistently led to lower estimated ORs and HRs for COVID-19-related death in cancer patients (e.g. any cancer versus no cancer; six studies, unadjusted OR=3.30,95%CI:2.59–4.20, adjusted OR=1.37,95%CI:1.16–1.61). Adjusted effect estimates were not reported for people with lung or haematological cancers. Of 18 studies that adjusted for at least age, 17 reported positive associations between pre-existing cancer diagnosis and COVID-19-related death (e.g. any cancer versus no cancer; nine studies, adjusted OR=1.66,95%CI:1.33–2.08; five studies, adjusted HR=1.19,95%CI:1.02–1.38).

**Conclusions:**

The initial evidence (published to 1 July 2020) on COVID-19-related death in people with cancer is characterised by multiple sources of bias and substantial overlap between data included in different studies. Pooled analyses of non-overlapping early data with adjustment for at least age indicated a significantly increased risk of COVID-19-related death for those with a pre-existing cancer diagnosis.

## Highlights

• Early studies on risk of COVID-19-related death and cancer have high risk of bias.
• There was substantial overlap between data included in different early studies.
• Meta-analyses suggest higher risk of COVID-19-related death for people with cancer.
• Stratified estimates by cancer type, time since diagnosis, and treatment are needed.

## 1 Introduction

The World Health Organisation declared COVID-19 (a disease caused by severe acute respiratory syndrome coronavirus 2 (SARS-CoV-2)) a pandemic on 11 March 2020. The pandemic has led to considerable disease burden worldwide, with > 182 million people infected and > 3.9 million attributed deaths by 1 July 2021 [1]. The early days of the pandemic were characterised by high uncertainty and an urgent need to understand who is most at risk of severe disease, to enable targeted shielding and precautionary measures. Initial reports suggested people with pre-existing conditions including cancer were at higher risk of death, with mechanistic hypotheses including effects of a compromised immune system due to cancer itself and/or cancer treatment [2]. Based on early studies, it was further proposed that lung cancer may increase risk due to concomitant lung damage, while haematological cancer may increase risk due to immunocompromise (secondary to the myelosuppressive nature of treatments and or impact of progression) [3], [4], [5], [6].

The pressing need for evidence to inform clinical practice and public health policy at the onset of the pandemic led to the rapid conduct, analysis and publication of studies under challenging circumstances. These were subsequently fast tracked for publication with or without formal peer review to facilitate real-time impact [7], [8]. However, multiple expressions of concern regarding the methodological quality of these studies were raised, including lack of adjustment for confounders, inadequate ascertainment of cancer status and other comorbidities [8], [9], [10]. Moreover, several studies were based on overlapping samples, leading to difficulties in determining which studies provided independent evidence [11].

The early reports have influenced clinical and policy decisions with respect to the cancer care pathway (e.g. delays in the delivery of or modification of cancer treatments) and increased anxiety for people with cancer and those who support them [12], [13], [14], [15], [16], [17], [18], [19], [20]. These effects could increase the burden of cancer, further to other pandemic-related impacts such as suspension of HPV vaccination, cancer screening programmes, as well as delays in diagnosis and treatment due to overwhelmed healthcare systems and redeployment of services [19], [21]. At different stages of the pandemic, decisions on the prioritisation of vaccine provision and the emergence of SARS-CoV-2 variants continue to require timely production and synthesis of high-quality evidence.

As the initial evidence played a critical role in decision-making during the pandemic, it is crucial to examine the strengths and limitations of that evidence and to gain insights for ongoing evidence reviews. Consequently, we carried out a systematic review of the early studies (to 1 July 2020) that provide information on the question, “Do COVID-19 patients with cancer have a higher risk of COVID-19-related death than those without cancer?”. We also carried out a separate systematic review, reported in a companion article, to examine whether people with cancer have higher risk of developing COVID-19. We identified, critically appraised and synthesised the results of early studies, focussing on sources of bias and methodological limitations, and the impact on the results.

## 2 Methods

The protocol for this systematic review was registered on PROSPERO (CRD42020191922).

### 2.1 Eligibility criteria

While we were particularly interested in COVID-19 mortality in cancer patients, because of potential limitations of cause-of-death coding and reporting in the first months of the pandemic, we broadened our inclusion criteria to include studies reporting COVID-19-specific or all-cause mortality after COVID-19 diagnosis (“COVID-19-related” death). Cohort and case-control studies were included if they reported COVID-19-related mortality for people with a previous diagnosis of cancer, compared to those who did not have a previous cancer diagnosis, or anyone with a COVID-19 diagnosis. Eligible exposures were previous diagnosis of any cancer, active cancer (cancer diagnosed or treated in the last year, or described as active), or specifically lung cancer or haematological cancer (based on biological hypotheses suggesting higher risks for people with these cancer types). Where the exposure was described as cancer with no further details provided, we classified this as “any cancer”. Studies restricted to populations with non-cancer-specific health conditions were excluded.

### 2.2 Information sources and search strategy

Medline and Embase databases were searched on 3 July 2020 for English-language articles published 1 January-1 July 2020 by combining database-specific subject headings and text terms for COVID-19 and cancer or comorbidities (Supplementary Table 1). Reference lists of relevant systematic reviews and full-text articles were checked for additional potentially relevant studies. All COVID-19-related pre-prints posted until 1 July 2020 on BioRxiv and MedRxiv (https://connect.biorxiv.org/relate/content/181) and the SSRN website (https://www.ssrn.com/index.cfm/en/coronavirus/) were also scanned.

### 2.3 Selection process

Two reviewers (CC or DC) screened titles and abstracts of identified published articles against pre-specified inclusion criteria with 10% assessed by both reviewers to ensure concordance. Titles and abstracts of pre-prints were screened by a single reviewer (SH). Full texts of potentially relevant articles were independently assessed for inclusion by two reviewers, with disagreements resolved by a third reviewer. Reasons for exclusion were recorded for all excluded full-text articles.

### 2.4 Data collection

Pairs of reviewers (chosen from CC, DC, VF, SH, HH, SY) independently extracted study characteristics and results for each included study, with disagreements resolved by third reviewer adjudication. The following information was extracted: publication status, study design, country, population characteristics, source of study population, study period, method of COVID-19 diagnosis, cancer definition and numbers, comparator definition and numbers, minimum possible length of follow-up, outcome definition, number of people with the outcome for those with and without pre-existing cancer and, where reported, the effect estimate and 95% confidence interval (95%CI) and any covariates included in analyses.

### 2.5 Risk of bias assessment

For each study included in the meta-analyses, risk of bias was independently assessed by a pair of reviewers (chosen from SA, CC, DC, VF, SH, DO’C, JS, SE), using modified versions of the Newcastle-Ottawa Scale designed specifically to assess the risk of bias in observational aetiological cohort and case-control studies (Supplementary Tables 2–3) [22]. Differences were resolved by consensus or adjudication by a third reviewer. The risk of bias was rated low, moderate or high for each of: selection of exposed and unexposed cohorts, co-interventions, exposure status ascertainment, reverse causation, outcome ascertainment, completeness and differences in follow-up, exclusions due to missing exposure or covariate data, adjustment for important confounders or over-adjustment, and the reliability of covariate data. As the full list of important confounders remains to be established, by definition, low risk of bias for this domain was not possible. Therefore, overall ratings for studies were limited to moderate risk of bias (low or moderate risk across all domains) or high risk (high risk for at least one domain). Studies were considered to have high risk of bias due to over-adjustment if they adjusted for an intermediate variable on the causal pathway between having cancer and death, e.g. the number of comorbidities including cancer or clinical indicators of COVID-19 severity.

### 2.6 Effect measures

Most studies reported the association between any pre-existing cancer diagnosis and death as adjusted and/or unadjusted odds ratios (ORs), risk ratios (RRs) or hazard ratios (HRs). Unadjusted rate ratios were calculated for studies with a general population comparator. If unadjusted effect estimates were not reported, we calculated ORs and 95%CIs from exposure-outcome cross tabulations with 0.5 added to each cell when there were zero cells [23].

### 2.7 Data synthesis

#### 2.7.1 Selection of studies for meta-analyses

To assess the impact of different comparisons, effect estimates, and adjustment for confounders on results, all analyses were conducted separately by combinations of effect measure, exposure measured, comparator, and study-type. Where a study reported the same effect estimate adjusted in more than one way, the effect estimate adjusted for the most covariates was selected unless there was a concern about over-adjustment.

To avoid data duplication, studies with overlapping samples were identified, and only the study with the largest number of people with cancer was included in a meta-analysis. Studies with insufficient or inconsistent data were excluded from the meta-analyses.

#### 2.7.2 Meta-analyses

Pooled effect estimates and 95%CIs from generic inverse-variance random-effects analyses were calculated using Stata 14. ORs and RRs were pooled together in the same meta-analysis as the risk of death was < 10% in both the cancer and comparison groups in all relevant studies [24]. If both ORs and RRs were available for a study, ORs were used as these were reported more often. HRs were pooled separately. To assess the effect of adjustment for confounders, we informally compared adjusted and unadjusted OR/RRs and HRs for studies where both were available (a statistical test was not possible as the estimates were obtained from the same studies).

#### 2.7.3 Assessment of heterogeneity

Heterogeneity was assessed with the χ^2^ test and I^2^ statistic (see Supplementary materials).

#### 2.7.4 Subgroup analysis and investigation of heterogeneity

For meta-analyses with sufficient numbers of studies, pre-specified subgroup analyses were performed for country, source of study population (general, hospitalised, hospitalised in ICU or with severe/critical disease), publication status (original journal article, pre-print) and covariates included in adjustment (age and sex only, >2 variables, over-adjusted). Possible subgroup differences were assessed using χ^2^ tests.

#### 2.7.5 Supplementary analyses

To assess the sensitivity of our primary results to our choice of analytical method, we repeated the two analyses with the most studies using fixed-effect rather than random-effects methods.

### 2.8 Reporting bias assessment

None of the meta-analyses of adjusted effect estimates included 10 or more studies, so we did not conduct pre-planned assessments of publication bias using visual inspection of funnel plot asymmetry and Egger's statistical test [25].

## 3 Results

**Fig. 1:**
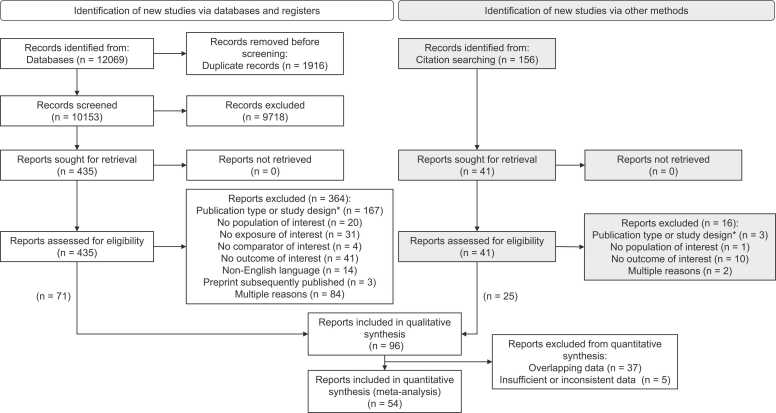
Flow diagram based on the PRISMA 2020 flow chart summarising the article screening process. * excluded publication type or study design, or letter or comment without relevant primary data.

In total, 12,225 records were identified and 96 studies satisfied the inclusion criteria (Fig. 1). The main reasons for exclusion were study design other than cohort or case-control study, or letter/comment without relevant data (Supplementary Table 4). Fifty-four studies [2], [4], [5], [6], [26], [27], [28], [29], [30], [31], [32], [33], [34], [35], [36], [37], [38], [39], [40], [41], [42], [43], [44], [45], [46], [47], [48], [49], [50], [51], [52], [53], [54], [55], [56], [57], [58], [59], [60], [61], [62], [63], [64], [65], [66], [67], [68], [69], [70], [71], [72], [73], [74], [75] were included in the meta-analyses after omitting 37 studies with overlapping samples and five studies with insufficient/inconsistent data to calculate effect estimates (Supplementary Table 5).

Characteristics of the 54 studies included in the meta-analyses are summarised in Table 1. These studies included over 500,000 people who developed COVID-19, of whom > 8000 had a pre-existing cancer diagnosis. Most studies were of hospital inpatients whose COVID-19 diagnosis was based on a SARS-CoV-2 PCR assay, and the minimum follow-up period was 0–30 days (0 days for the majority of studies). Four studies specifically reported deaths from COVID-19 or due to acute respiratory distress, while all other studies reported overall mortality only.

**Table 1:** Characteristics of studies included in meta-analyses.

Study	Publication type	Population					Exposure (Cancer)			Comparator		Minimum follow up (days)
		Description	N	Age (median years)	Male (%)	Method of COVID-19 diagnosis	Cancer status	Cancer type	n	Definition	n	
**24 countries**												
COVIDSurg Collaborative^ [35]	Original journal article	Surgical patients	823	NR	NR	PCR assay or clinical/ imaging	Active	NR	239	No active cancer	584	29
**China**												
Chen M [33]	Preprint	Hospital inpatients	123	NR	49.6	PCR assay	NR	NR	10	No cancer	113	NR
Guan W [39]	Original journal article	Hospital inpatients	1590	48.9^a^	57.3	PCR assay	NR	NR	18	No cancer	1572	0
He W [4]	Original journal article	Hospital inpatients	24	35 (cancer) 32 (non-cancer)	37.5	PCR assay or clinical/ imaging	Any	Haematological	13	No cancer	11	15
Li J^^ [47]	Preprint	Hospital inpatients	161	NR	49.7	PCR assay	NR	NR	2	No cancer	159	0
Liu M [48]	Preprint	Hospital inpatients	665	58	47.8	PCR assay	NR	NR	18	No cancer	647	0
Liu Y [49]	Original journal article	Hospital inpatients	245	54.0^a^	46.5	PCR assay	NR	NR	9	No cancer	236	0
Meng Y [6]	Original journal article	Hospital inpatients	2665	62 (cancer) 58 (non-cancer)	49.8	PCR assay	Any	Mixed	109	No cancer	2556	0
			2572	NR	NR	PCR assay	Any	Haematological	16	No cancer	2556	0
			2570	NR	NR	PCR assay	Active	Mixed	14	No cancer	2556	0
NCPERET^b^[2]	Original journal article	All COVID-19 patients	44,672	NR	51.4	PCR assay	NR	NR	107	All COVID cases in a population	44,672	0
Shi S [67]	Original journal article	Hospital inpatients with severe disease	671	63	48.0	PCR assay	NR	NR	23	No cancer	648	0
Tian J [69]	Original journal article	Hospital inpatients	751	64	50.0	PCR assay	Any	Mixed	232	No cancer	519	7
			542	NR	NR	PCR assay	NR	Lung	23	No cancer	519	7
Wang K [70]	Original journal article	Hospital inpatients	296	47.3^a^	47.3	PCR assay or clinical/imaging	NR	NR	1	No cancer	295	2
Wang L [71]	Original journal article	Hospital inpatients	339	69	48.9	PCR assay	NR	NR	15	No cancer	324	28
Xie J [73]	Preprint	Hospital inpatients with critical disease	733	65	65.1	PCR assay or serology	NR	NR	24	No cancer	709	28
Yao Q [74]	Original journal article	Hospital inpatients	108	52	39.8	PCR assay	NR	NR	2	No cancer	106	20
Zhao M [75]	Original journal article	Hospital inpatients	1000	61	46.6	PCR assay	NR	NR	28	No cancer	972	9
**Denmark**												
Reilev M [61]	Preprint	All COVID-19 patients	9519	49	42.1	PCR assay	NR	NR	735	No cancer	8784	14
**France**												
Luong-Nguyen M [50]	Original journal article	Hospital inpatients	15	62	60.0	PCR assay	NR	Mixed	10	No cancer	5	0
**Iran**												
Abdolahi N [26]	Preprint	Hospital inpatients	332	NR	NR	Unclear	NR	NR	4	No cancer	328	30
Javanian M [43]	Preprint	Hospital inpatients	100	60.1^a^	51.0	PCR assay	NR	NR	4	No cancer	96	4
Nikpouraghdam M [56]	Original journal article	Hospital inpatients	2964	56	66.0	PCR assay or clinical/imaging	NR	NR	17	No cancer	2947	0
Shahriarirad R [66]	Original journal article	Hospital inpatients	113	53.8^a^	62.8	PCR assay	NR	NR	1	No cancer	112	0
**Italy**												
Benelli G [30]	Preprint	Hospital inpatients and attendees	411	71	Unclear	PCR assay	Any	NR	33	No cancer	378	6
Borghesi A [31]	Original journal article	Hospital inpatients	302	67	64.2	PCR assay	Any	NR	56	No cancer	246	0
Ciceri F [34]	Original journal article	Hospital inpatients	405	NR	NR	PCR assay	Active	NR	22	No active cancer	383	30
Masetti C [52]	Original journal article	Hospital inpatients	229	60.7^a^	64.6	PCR assay	NR	NR	24	No cancer	205	0
Montopoli M [55]	Original journal article	All COVID-19 patients	4532	NR [68.1% age < 70 y]	100	PCR assay	NR	Mixed	430	No cancer	4102	0
Rossi P [64]	Preprint	Symptomatic patients	2362	NR	NR	PCR assay	NR	NR	301	No cancer	2061	0
Stroppa E [68]	Original journal article	Hospital inpatients	56	NR	62.5	PCR assay	NR	Mixed	25	No cancer	31	0
			43	NR	NR	PCR assay	Active	NR	12	No cancer	31	0
			39	NR	NR	PCR assay	NR	Lung	8	No cancer	31	0
			33	NR	NR	PCR assay	NR	Haematological	2	No cancer	31	0
**Korea**												
Lee H [46]	Preprint	All COVID-19 patients	8266	45	38.5	PCR assay	NR	NR	364	No cancer	7902	7
**Poland**												
Nowak B [57]	Original journal article	Hospital inpatients	169	63.7^a^	51.5	PCR assay	NR	NR	35	No cancer	134	0
**Portugal**												
Peixoto V [59]	Preprint	All COVID-19 patients	20,270	NR [34.8% age ≥ 60 y]	41.3	PCR assay	NR	NR	603	No cancer	19,667	0
**Spain**												
Borobia A [32]	Original journal article	Hospital inpatients	2226	61	48.2	Unclear	NR	Mixed	385	No cancer	1841	0
			1974	NR	NR	Unclear	NR	Haematological	133	No cancer	1841	0
Heili-Frades S [40]	Preprint	Hospital inpatients and attendees	4712	62.0^a^	49.4	PCR assay	NR	NR	239	No cancer	4473	0
Iftimie S^ [41]	Preprint	Hospital inpatients	188	66.4^a^	55.8	PCR assay	NR	NR	26	No cancer	162	0
Lara Alvarez M [45]	Original journal article	Hospital inpatients	1069	NR	NR	PCR assay	Any	Mixed	36	No cancer	1033	0
Perez-Tanoira A [60]	Preprint	Hospital inpatients and attendees	392	71	52.6	PCR assay	NR	NR	53	No cancer	339	18
Rogado J [63]	Original journal article	All COVID-19 patients	42,450	NR	NR	Unclear	Any	Mixed	45	All COVID-19 cases in a population	42,450	0
**UK**												
Aries J [28]	Letter - peer reviewed	All COVID-19 patients	223,060	NR	NR	PCR assay	Any	Haematological	35	All COVID-19 cases in a population	223,060	14
			223,060	NR	NR	PCR assay	Active	Haematological	24	All COVID-19 cases in a population	223,060	14
Docherty A^ [36]	Original journal article	Hospital inpatients	17,354	NR	60	PCR assay	NR	NR	1743	No cancer	15,611	14
Galloway J [37]	Original journal article	Hospital inpatients	1156	NR	57.6	PCR assay	Active	NR	118	No active cancer	1038	0
Joharatnam-Hogan N [44]	Preprint	Hospital inpatients	52	76 (Cancer) 59 (Non-cancer)	59.6	PCR assay	Active	Mixed	26	No cancer	26	0
Sapey E [65]	Preprint	Hospital inpatients	2217	73	58.2	PCR assay	Active	NR	152	No active cancer	2065	24
**USA**												
Aggarwal S [27]	Original journal article	Hospital inpatients	16	67	75	PCR assay	NR	NR	3	No cancer	13	0
Azar K [29]	Original journal article	All COVID-19 patients	1052	53.0^a^	49.2	Unclear	NR	NR	55	No cancer	997	0
Garibaldi B [38]	Preprint	Hospital inpatients	832	63	53.2	PCR assay	NR	NR	90	No cancer	742	10
Imam Z [42]	Original journal article	Hospital inpatients	1305	61.0^a^	53.8	PCR assay	NR	NR	83	No cancer	1222	15
Marcello R [51]	Preprint	Hospital inpatients	6200	NR	NR	PCR assay	Any	NR	601	No cancer	5599	6
Mehta V [5]	Original journal article	All COVID-19 patients	104,185	50	53.3	Unclear	NR	Haematological	54	All COVID-19 cases in a population	104,185	0
			104,185	50	53.3	Unclear	NR	Lung	11	All COVID-19 cases in a population	104,185	0
			104,185	50	53.3	Unclear	Active	Mixed	92	All COVID-19 cases in a population	104,185	0
		Symptomatic patients	1308	NR	NR	PCR assay	Mixed	Mixed	218	No cancer	1090	3
			1144	NR	NR	PCR assay	NR	Haematological	54	No cancer	1090	3
			1101	NR	NR	PCR assay	NR	Lung	11	No cancer	1090	3
			1182	NR	NR	PCR assay	Active	Mixed	92	No cancer	1090	3
Mendy A [53]	Preprint	All COVID-19 patients	689	50	53.0	PCR assay	Any	NR	136	No cancer	553	0
Miyashita H [54]	Letter or commentary	Hospital inpatients and attendees	5688	NR	NR	PCR assay	NR	Mixed	334	No cancer	5354	2
Palaiodimos L [58]	Original journal article	Hospital inpatients	200	64	49.0	Unclear	Active	NR	11	No active cancer	189	20
Robilotti E [62]	Original journal article	All COVID-19 patients	176,086	51	51.4	Unclear	NR	Mixed	963	All COVID-19 cases in a population	176,086	0
Wang Z [72]	Preprint	Hospital inpatients	3273	NR	57.3	PCR assay	Active	NR	233	No active cancer	3040	1

NR = not reported; PCR = Polymerase chain reaction.

All retrospective cohort studies except 3 prospective cohort studies (^) and 1 nested case-control study (^^).

a Mean.

b Novel Coronavirus Pneumonia Emergency Responses Epidemiology Team.

Of the studies included in meta-analyses, 19 provided information on cancer status (e.g. active or not) and 11 specifically restricted analyses to active cancer. Fifty-two studies either did not specify cancer type or only reported cancer type for sub analyses (three reported on lung cancer and four on haematological cancers) (Table 1). Two studies included only haematological cancers. Details and results for the 17 meta-analyses conducted are shown in Table 2.

**Table 2:** Numbers of studies and people, and pooled effect estimates for each meta-analysis.

Analysis^a^	Exposure group	Comparison group	Measure of effect	Number of studies	People with cancer alive	People with cancer dead	Comparator alive	Comparator dead	Total^b^	Pooled effect estimate (95%CI)	I^2^ (p-het)
1^c^	Any cancer	No cancer	Unadjusted OR	33	4839	1542	78,786	9653	94,820	2.54 (2.06,3.13)	83% (<0.001)
2	Any cancer	No cancer	Adjusted OR/RR	9	1864	403	33,866	1752	37,885	1.66 (1.33,2.08)	51% (0.038)
3	Any cancer	No cancer	Unadjusted HR	2	1244	483	12,763	3022	17,512	1.56 (1.43,1.70)	0% (0.434)
4	Any cancer	No cancer	Adjusted HR	5	1964	465	23,073	4032	29,534	1.19 (1.02,1.38)	16% (0.312)
5^c^	Any cancer	No cancer	Unadjusted OR	1	0	2	96	63	161	6.10 (0.27,137.49)	n/a
6	Any cancer	Any COVID diagnosis	Unadjusted rate ratio	3	1003	112	236,897	26311	263,208^b^	1.82 (0.63,5.24)	96% (<0.001)
7	Active cancer	No cancer	Unadjusted OR	5	106	46	3287	421	3860	2.77 (1.88,4.09)	0% (0.452)
8	Active cancer	No active cancer	Unadjusted OR	4	270	148	4162	1515	6095	1.58 (1.11,2.25)	48% (0.125)
9	Active cancer	No active cancer	Adjusted OR	1	173	66	455	129	823	1.55 (1.01,2.39)	n/a
10	Active cancer	No active cancer	Unadjusted HR	1	11	11	302	81	405	2.77 (1.47,5.22)	n/a
11	Active cancer	No active cancer	Adjusted HR	3	270	103	3470	991	4834	1.25 (0.90,1.73)	51% (0.128)
12	Active cancer	Any COVID diagnosis	Unadjusted rate ratio	1	60	32	98,003	6182	104,185^b^	5.86 (4.43,7.76)	n/a
13	Haematological cancers	No cancer	Unadjusted OR	5	134	84	4793	736	5747	4.60 (2.38,8.86)	61% (0.037)
14	Haematological cancers	Any COVID diagnosis	Unadjusted rate ratio	2	55	34	288,998	38247	327,245^b^	4.19 (1.90,9.26)	89% (0.003)
15	Active haematological	Any COVID diagnosis	Unadjusted rate ratio	1	15	9	190,995	32065	223,060^b^	2.61 (1.17,5.84)	n/a
16	Lung cancer	No cancer	Unadjusted OR	3	25	17	1430	210	1682	5.13 (2.70,9.73)	0% (0.384)
17	Lung cancer	Any COVID diagnosis	Unadjusted rate ratio	1	5	6	98,003	6182	104,185^b^	9.19 (5.36,15.77)	n/a
		**Total across all analyses**^d^**:**		**80**	**12,038**	**3563**	**1079,379**	**131,592**	**1225,241**		

Numbers were estimated for studies that did not report specific numbers of deaths and non-deaths, using total numbers in cancer and comparator groups and reported effect estimates.

a Forest plots for the meta-analyses are shown in Fig. 2, Fig. 3, Fig. 4 and Supplementary Figures 1–18, including additional subgroup analyses and sensitivity analyses using fixed effects rather than random effects for Analyses 1 and 2. For Analyses 1 and 2, subgroup analyses were performed by country, source of population (general, hospitalised, hospitalised in ICU or with severe/critical disease), and publication status (published, pre-print). For Analysis 2, additional subgroup analyses were also performed by adjustment (adjusted age and sex only, adjusted for > 2 factors, over-adjusted).

b For studies where the comparator is “Any COVID diagnosis”, people with cancer in the exposure group are a subset of the comparator group; however, these people are only counted once in the total.

c Analysis 1 included 33 cohort studies while analysis 5 included one nested case-control study.

d Totals include multiple counts of the same studies and people included in different analyses.

All 54 studies had high risk of bias (Table 3, Table 4). The main sources of bias were unclear or inadequate ascertainment of cancer status, potential differences in treatment or management of COVID-19 patients with and without cancer, limited or lacking ascertainment of confounders, and insufficient control for important confounders. Adjustment for important confounders was assessed to be at moderate risk of bias for the 15 of 18 studies that controlled for at least age (adjustments used in individual studies are listed in Supplementary Table 6).

**Table 3:** Risk of bias of cohort studies included in analyses.

Study	1	2	3	4	5	6	7	8	9a	9b	9c	Overall rating
COVIDSurg Collaborative [35]	Low	High	Low	Low	Low	Low	High	Low	Moderate	High	High	High
Chen M [33]	Low	High	High	Low	Low	High	High	Moderate	High	NA	NA	High
Guan W [39]	Low	High	High	Low	Low	Low	Low	Low	High	NA	NA	High
He W [4]	Low	High	High	Low	Low	Low	Low	High	High	NA	NA	High
Liu M [48]	Low	High	High	Low	Low	Moderate	Low	Low	High	NA	NA	High
Liu Y [49]	Low	High	High	Low	Low	High	High	Low	High	NA	NA	High
Meng Y [6]	Low	High	High	Low	Low	Moderate	High	Low	High	NA	NA	High
NCPERET^a^[2]	Low	High	High	Low	Low	High	High	Low	High	NA	NA	High
Shi S [67]	Low	High	High	Low	Low	Low	Low	High	High	NA	NA	High
Tian J [69]	Low	Low	High	Low	Low	Low	Low	Low	Moderate	Low	Low	High
Wang K [70]	Low	High	High	Low	Low	High	High	Low	High	NA	NA	High
Wang L [71]	Low	High	High	Low	Low	Low	Low	Low	High	NA	NA	High
Xie J [73]	Low	Low	High	Low	Low	Low	Low	Low	Moderate	High	High	High
Yao Q [74]	Low	High	High	Low	Low	Low	Low	Low	High	NA	NA	High
Zhao M [75]	Low	High	High	Low	Low	Low	Low	High	High	High	High	High
Reilev M [61]	Low	High	Low	Low	Low	Low	Low	Low	Moderate	NA	NA	High
Luong-Nguyen M [50]	Low	High	High	Low	Low	Low	Low	Low	High	NA	NA	High
Abdolahi N [26]	Low	High	High	Low	Low	Moderate	High	High	High	NA	NA	High
Javanian M [43]	Low	High	High	Low	Low	High	High	Low	High	NA	NA	High
Nikpouraghdam M [56]	Low	High	High	Low	Low	Low	Low	Low	High	NA	NA	High
Shahriarirad R [66]	Low	High	High	Low	Low	Low	Low	Low	High	NA	NA	High
Benelli G [30]	Low	High	High	Low	Low	High	High	Low	High	NA	NA	High
Borghesi A [31]	Low	High	High	Low	Low	High	High	High	High	NA	NA	High
Ciceri F [34]	Low	High	High	Low	Low	Low	Low	Low	Moderate	Low	Moderate	High
Masetti C [52]	Low	High	High	Low	Low	High	High	High	High	NA	NA	High
Montopoli M [55]	Low	High	Low	Low	Low	High	High	Low	High	NA	NA	High
Rossi P [64]	Low	High	Low	Low	Low	Low	Low	High	Moderate	NA	NA	High
Stroppa E [68]	Low	Low	High	Low	Low	Low	Low	Low	Moderate	High	High	High
Lee H [46]	Low	High	Low	Low	Low	Low	Low	Low	Moderate	Low	Low	High
Nowak B [57]	Low	High	High	Low	Low	Low	Low	Low	High	NA	NA	High
Peixoto V [59]	Low	High	High	Low	Low	Low	Low	Low	Moderate	Low	Low	High
Borobia A [32]	Low	High	High	Low	Low	Moderate	High	Moderate	High	NA	NA	High
Heili-Frades S [40]	Low	High	High	Low	Low	High	High	High	High	High	Low	High
Iftimie S [41]	Low	High	High	Low	Low	Low	Low	High	Moderate	High	Low	High
Lara Alvarez M [45]	Low	High	High	Low	Low	Low	Low	Low	High	NA	NA	High
Perez-Tanoira A [60]	Low	High	High	Low	Low	Moderate	High	Low	High	High	Moderate	High
Rogado J [63]	High	High	Moderate	Low	Low	Low	High	Low	High	NA	NA	High
Aries J [28]	High	High	Moderate	Low	High	High	High	Low	High	NA	NA	High
Docherty A [36]	Low	High	High	Low	Low	Low	Low	Moderate	Moderate	High	Low	High
Galloway J [37]	Low	High	High	Low	Low	Low	Low	Low	Moderate	NA	NA	High
Joharatnam-Hogan N [44]	Low	High	High	Low	Low	Low	Low	Low	High	NA	NA	High
Sapey E [65]	Low	High	High	Low	Low	Low	Low	Low	High	NA	NA	High
Aggarwal S [27]	Low	High	High	Low	Low	Low	Low	Low	High	NA	NA	High
Azar K [29]	Low	High	Moderate	Low	Low	High	High	Moderate	High	NA	NA	High
Garibaldi B [38]	Low	High	Moderate	Low	Low	Low	Low	Moderate	High	NA	NA	High
Imam Z [42]	Low	High	Low	Low	Low	Low	Low	High	High	NA	NA	High
Marcello R [51]	Low	High	Moderate	Low	Low	Low	Low	Low	High	NA	NA	High
Mehta V [5]	High	High	Moderate	Low	Low	Low	Low	Low	Moderate	NA	NA	High
Mendy A [53]	Low	High	Moderate	Low	Low	High	High	Low	Moderate	Low	Low	High
Miyashita H [54]	Low	High	Low	Low	Low	High	High	Low	High	NA	NA	High
Palaiodimos L [58]	Low	High	High	Low	Low	Low	Low	Low	High	NA	NA	High
Robilotti E [62]	High	High	Moderate	Low	High	High	High	Low	High	NA	NA	High
Wang Z [72]	Low	High	Moderate	Low	Low	Low	Low	Low	Moderate	Low	Moderate	High

1 = Exposed and comparison (unexposed) populations and selection of cohort(s); 2 = Similarity of co-interventions between groups; 3 = Nature and measurement of exposure; 4 = Timing of exposure measurement and outcome (reverse causation) 5 = Nature and measurement of outcome; 6 = Completeness of follow up; 7 = Differences in follow up; 8 = Exclusions due to missing data on other variables; 9a = Control of confounding: Comparability of exposed and unexposed cohorts with respect to potentially important confounding variables; 9b = Control of confounding: reliability of assessment of presence or absence of prognostic factors 9c = Control of confounding: Covariates are appropriately included in the analysis; NA = not applicable.

a Novel Coronavirus Pneumonia Emergency Responses Epidemiology Team.

**Table 4:** Risk of bias of nested case-control study included in analyses.

Study	1	2	3	4	5	6	7	8	9	10	11	12	13	14	15	Overall rating
Li J [47]	Low	High	Low	Low	Low	High	Low	Low	Low	NA	High	High	NA	NA	NA	High

1 = Sources of cases (deaths) and controls (survivors); 2 = Selection of cases and controls; 3 = Definition of cases (outcome); 4 = Definition of controls; 5 = Timing of outcome of interest and exposure measurement (reverse causation); 6 = Measurement of exposure (pre-existing cancer); 7 = Method used to measure exposure (cancer) in cases and controls; 8 = Completeness of follow-up of cohort; 9 = Difference in follow-up between exposed and unexposed members of cohort; 10 = Exclusions due to missing data on other variables; 11 = Comparability of cases and controls on important confounding variables; 12 = Similarity of co-interventions between groups; 13 = Assessment of the presence or absence of prognostic factors; 14 = Covariates are appropriately included in statistical analysis models; 15 = Analysis appropriate to design; NA = not applicable.

**Fig. 2:**
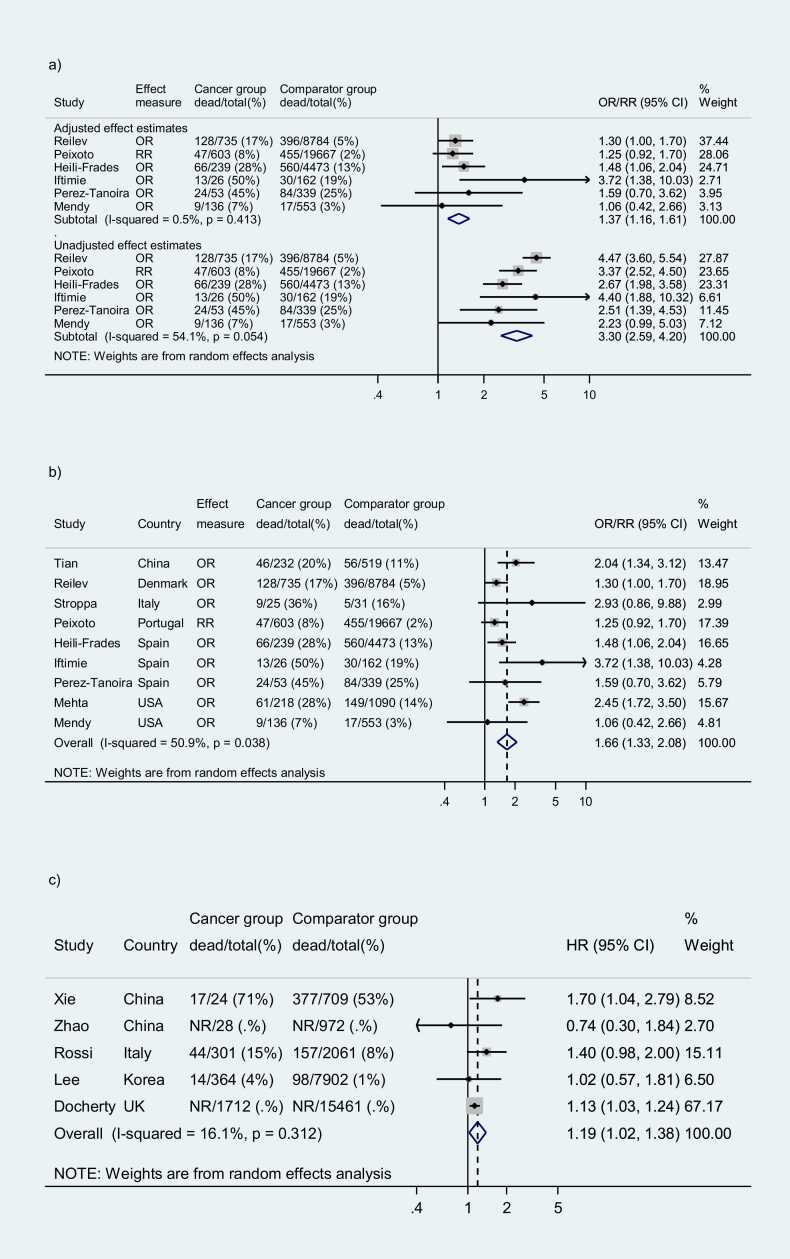
Adjusted effect estimates for COVID-19-related death for people with a pre-existing diagnosis of any cancer, compared to those without. a) Comparison of unadjusted ORs or RRs and estimates at least adjusted for age, from 6 studies that presented data for both, b) Meta-analysis of 9 studies that reported adjusted ORs or RRs (Analysis 2 in Table 2), c) Meta-analysis of 5 studies that reported adjusted HRs (Analysis 4 in Table 2), Whiskers represent 95% CIs. Estimates > 1 represent higher risk of COVID-19-related death for people with a pre-existing cancer diagnosis.

In a comparison of unadjusted and adjusted ORs or RRs in six studies (Fig. 2a), the pooled adjusted effect estimate for pre-existing cancer and COVID-19-related death was lower than the corresponding unadjusted estimate (adjusted OR/RR=1.37, 95%CI:1.16–1.61; unadjusted OR/RR=3.30, 95%CI:2.59–4.20). This was also observed for one study reporting hazard ratios (adjusted HR=1.13, 95%CI:1.03–1.24; unadjusted HR=1.56, 95%CI:1.43–1.70), and when comparing pooled estimates from all studies reporting unadjusted and all studies reporting corresponding adjusted estimates (Analyses 1–4, 8–11 in Table 2).

**Fig. 3:**
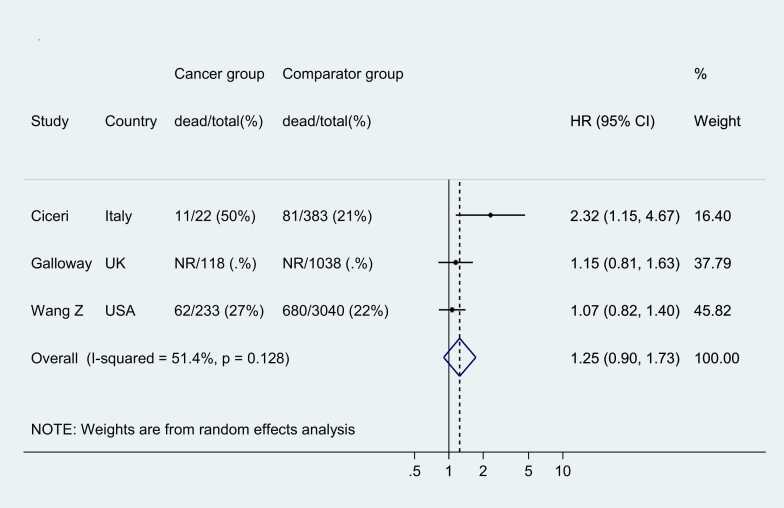
Adjusted HRs for COVID-19-related death for people with active cancer, compared to those without active cancer. Meta-analysis of 3 studies that reported adjusted HRs (Analysis 11 in Table 2). Whiskers represent 95% CIs. Estimates > 1 represent higher risk of COVID-19-related death for people with active cancer.

Given these differences, we focused the interpretation of results on studies that adjusted or controlled for at least age. Of these 18 studies, 17 reported effect estimates indicating a positive association between pre-existing cancer and COVID-19-related death (including 15 studies with non-overlapping patient groups, see Supplementary appendix). People with a pre-existing diagnosis of cancer had significantly higher risk of death (nine studies, adjusted OR/RR=1.66, 95%CI:1.33–2.08; moderate heterogeneity *I*^*2*^=51%, p = 0.038; Fig. 2b, Analysis 2). The results were similar for pooled adjusted hazard ratios (five studies, adjusted HR=1.19, 95%CI:1.02–1.38; low heterogeneity *I*^*2*^=16%, p = 0.312; Fig. 2c, Analysis 4). The results were also similar for the comparison of people with active and no active cancer (one study, adjusted OR=1.55, 95%CI:1.01–2.39; three studies, adjusted HR=1.25, 95%CI:0.90–1.73; Fig. 3, Analyses 9 and 11).

None of the studies of haematological or lung cancers adjusted for age (Table 2 shows unadjusted effect estimates).

**Fig. 4:**
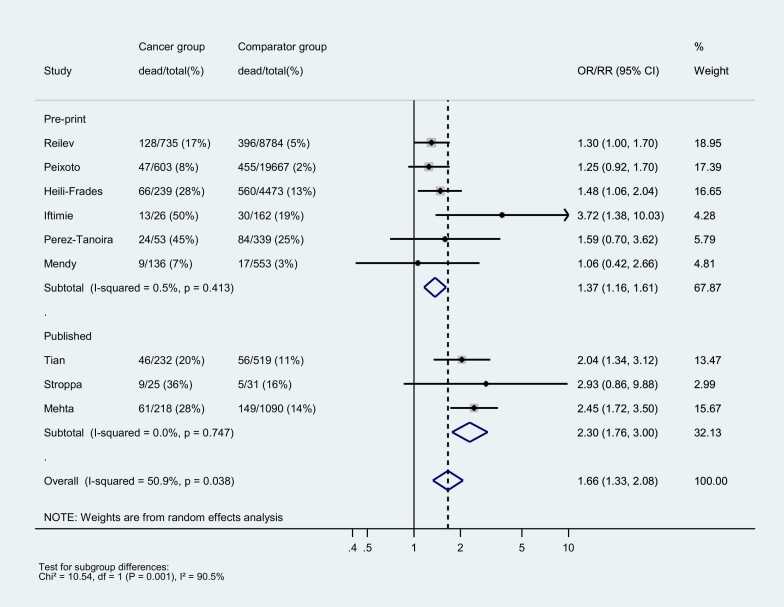
Adjusted ORs or RRs for COVID-19-related death for people with a pre-existing diagnosis of any cancer compared to those without, by publication status. Meta-analysis of studies that reported adjusted ORs or RRs (Analysis 2 in Table 2, grouped by publication status): “pre-print” denotes pre-print articles not published by 1 July 2020. Estimates > 1 represent higher risk of COVID-19-related death for people with a pre-existing cancer diagnosis.

Subgroup analyses to examine sources of heterogeneity were possible for nine studies that adjusted or controlled for age and reported ORs or RRs comparing any cancer to no cancer (Fig. 4; Supplementary Figures 6–8). Pooled effect estimates were significantly higher (p = 0.001) for studies published up to 1 July 2020 (adjusted OR/RR=2.30, 95%CI:1.76–3.00) than for pre-prints (adjusted OR/RR=1.37, 95%CI:1.16–1.61). Notably, the included published studies all controlled for age by matching people with and without cancer, while all pre-print studies adjusted for age in the analyses. There were no significant differences when stratified by country (p = 0.28), covariates included in the adjustment (p = 0.84), or source of study population (p = 0.06). Only one of these nine studies reported estimates for COVID-19 as cause of death (as opposed to overall mortality), so we could not assess heterogeneity due to specific cause of death. We verified that the pooled estimate for these nine studies was similar when using a fixed-effect instead of random effect meta-analysis (pooled fixed-effect OR/RR=1.58, 95%CI:1.37–1.81; Supplementary Figure 18).

## 4 Discussion

Our systematic review synthesised early evidence on the risk of COVID-19-related death for people with cancer from 54 studies reporting on > 500,000 COVID-19 cases with > 60,000 deaths (including >1800 deaths for people with pre-existing cancer). Of the 96 studies that satisfied inclusion criteria, 37 studies included patients who were also included in other larger studies, complicating the identification of independent evidence. Many studies had short follow-up periods, a small number of people with cancer, and unclear definitions of cancer status. All 54 studies included in meta-analyses had high risk of bias, the majority with multiple sources of bias, leading to uncertainty regarding the strength of the association between pre-existing cancer and risk of COVID-19-related death. Only 18 studies adjusted effect estimates for at least age. Even minimally adjusted effect estimates were consistently smaller than the corresponding unadjusted estimates. Nonetheless, pooled adjusted estimates indicated a significantly increased risk of death for those with pre-existing cancer.

In the early stages of the pandemic, based on the precautionary principle, concerns regarding the risk of severe COVID-19 and death for people with cancer who are exposed to SARS-CoV-2 led to cancer treatment protocol changes in different countries and settings. However, treatment changes may also lead to cancer progression and death, so early high-quality evidence on the magnitude of risk is imperative. We found the early evidence was largely limited, and early systematic reviews were confined to unadjusted effect estimates and small sample sizes, and did not assess risk of bias for individual studies [76], [77], [78], [79], [80], [81], [82], [83]. Our systematic review has provided in-depth critical assessment of the evidence generated early in the COVID-19 pandemic that can inform the design of future studies and ongoing reviews, but has some limitations. The titles and abstracts were not screened independently by two reviewers, although there was high agreement for the subset screened in duplicate. We did not contact the authors of included studies to clarify missing or unclear details, or to obtain additional information. For the risk of bias assessment, we could not identify all important confounders, as definitive evidence for conditions and characteristics associated with COVID-19-related death is yet to emerge. Given the heterogeneity in definitions of disease severity, we focussed on death as the outcome of interest, rather than more broadly and inconsistently defined severe COVID-19. However, our work also has several strengths including a comprehensive assessment of the early evidence, a focus on effect estimates adjusted at least for age, and in-depth risk of bias assessment.

We note that most of the early studies reported deaths from any cause after COVID-19 diagnosis, rather than COVID-19 specific deaths. Although cancer deaths could contribute to the elevated risk of death for COVID-19 patients with a pre-existing cancer diagnosis compared to those without, the contribution is likely be small given the short follow-up periods (usually two months or less) in the early studies. The World Health Organisation guidelines further specify that “A death due to COVID-19 is defined … as a death resulting from a clinically compatible illness, in a probable or confirmed COVID-19 case, unless there is a clear alternative cause of death that cannot be related to COVID disease (e.g. trauma). … A death due to COVID-19 may not be attributed to another disease (e.g. cancer) and should be counted independently of pre-existing conditions that are suspected of triggering a severe course of COVID-19” [84]. This is consistent with our analysis strategy in which all deaths of people with COVID-19 were treated as COVID-19-related. Later studies also considered COVID-19-related death as any death with COVID-19 mentioned on the death certificate (e.g. OpenSAFELY) [85], or confirmed/suspected COVID-19 deaths as described on the death certificate together with all deaths occurring in individuals with confirmed SARS-CoV-2 infection in the initial study period (e.g. QCOVID) [86].

While the early studies did not carry out in-depth analyses of time since cancer diagnosis or receipt of specific cancer treatments, several larger studies published after 1 July 2020 (which were also able to adjust for multiple potential confounders) suggest that both factors may increase the risk of COVID-19-related death. For example, the OpenSAFELY study, with > 17 million general practice patients in the UK and > 10,000 COVID-19 deaths [85], reported that diagnosis of non-haematological cancers closer to COVID-19 development was associated with higher risk of death, with no association for cancers diagnosed 5 + years prior to developing COVID-19 (cancer compared to no cancer: fully adjusted HR 1.72 (95%CI:1.50–1.96), 1.15 (95%CI:1.05–1.27) and 0.96 (95%CI:0.91–1.03) for cancers diagnosed < 1 year, 1–4.9 years, and 5 + years previously, respectively). Consistent with prior biological hypotheses, increased risk was larger for haematological cancers: fully adjusted HR 2.80 (95%CI:2.08–3.78), 2.46 (95%CI:2.06–2.95), 1.61 (95%CI:1.39–1.87) for cancers diagnosed < 1 year, 1–4.9 years, and 5 + years previously, respectively. The QCOVID study in the UK also reported significant associations between risk of COVID-19-related death and receipt of chemotherapy in the previous 12 months (women: adjusted HR 2.30 (95%CI:1.35–3.94), 3.52 (95%CI:2.29–5.42) and 17.31 (95%CI:6.52–45.98) for chemotherapy grade A, B, and C, respectively; men: adjusted HR 1.74 (95%CI:1.10–2.75), 3.50 (95%CI:2.54–4.82) and 4.47 (95%CI:1.17–9.64) for chemotherapy grade A, B, and C, respectively; where chemotherapy grades A, B, and C are based on risk of Grade 3/4 febrile neutropenia (CTCv4) or lymphopenia of < 10%, 10–50% and > 50%, respectively) [86]. This study also reported a positive association between risk of death from COVID-19 and radiotherapy receipt in the previous 6 months (women: adjusted HR 2.11 (95%CI:1.30–3.41); men: adjusted HR 2.09 (95%CI:1.48–2.96)).

As our systematic review focused on a critical appraisal of early evidence, the methodological insights gained from this review will inform the COVID-19 and Cancer Global Modelling Consortium (CCGMC; CCGMC.org) Observatory platform for the ongoing review and analysis of emerging data on risk of COVID-19-related death for people with cancer, supporting efforts for timely identification and synthesis of high-quality evidence. As part of the CCGMC efforts, the Observatory will help provide informed advice to support international decision-making in cancer control both during and after the pandemic, and we will aim to keep this review up-to-date as a living review. The Observatory will also include modelled estimates of COVID-19 impact, and the evidence from this review provisionally supports taking into account potential accelerated mortality in cancer patients with COVID-19, although more data are needed by cancer type and stage.

Recently, concerns regarding the risk of severe COVID-19 and death for people with cancer who are exposed to SARS-CoV-2 led multiple organisations to call for people with cancer to be prioritised for vaccination, including the American Association for Cancer Research, the National Comprehensive Cancer Network, and the European Society for Medical Oncology [87], [88], [89], [90]. As the application of the precautionary principle for all high-risk groups only provides limited information, ideally, decisions on prioritisation would be based on a nuanced understanding of risks for different subgroups of the population, including risks for people with cancer depending on time since diagnosis, treatment type and time since treatment. Comparisons to other individuals at high risk would also be needed to evaluate the trade-offs in prioritising specific groups.

In the future, vaccination efforts in many countries may reduce the risks posed by COVID-19, but the emergence of several SARS-CoV-2 variants of concern will require ongoing monitoring of disease risk [91]. At the time of writing, there was little direct evidence on vaccine effectiveness in specific subpopulations of people with cancer. To enable assessments of vaccine effectiveness for people with cancer, the availability of immunisation registers and linkage to cancer registries, medical and death records will be important. As for analyses of COVID-19 outcomes, this would ideally also include well-powered subgroup analyses by cancer type, treatment type, presence of other/specific comorbidities, and time since diagnosis and treatment. Linkage to comprehensive medical records would also facilitate adjustment for important confounders such as age and comorbidities, acknowledging that the recording of key covariates may still be incomplete, and some important factors (e.g. ethnicity) may be difficult to ascertain from routinely collected data. Thus, enhanced data collection for suitable surveillance cohorts is important. As provision of real-time information remains a challenge for many population-wide registries, to enable rapid and evidence-based responses to emerging variants, investments in infrastructure are needed to ensure high-quality near-time record linkage and accurate yet timely assessments of health impacts.

In conclusion, the early literature on risk of COVID-19-related death for people with cancer was characterised by pervasive biases and analytical limitations. Data from analyses adjusted at least for age suggest a higher risk of COVID-19-related death for people with cancer. Fine-grained analyses of surveillance cohorts of cancer patients and population-wide record linkage including cancer and immunisation registries, and real-time availability of clinical information will be important to inform the ongoing public health response to the COVID-19 pandemic.

## Funding

No specific funding was received for this study. Professor Karen Canfell receives salaray support from the National Health and Medical Research Council Australia (APP1194679).

## Author contributions

KC and D’OC conceived the study. KC, D’OC, JS, SE, SH, VF, CC, DC designed the study. VF, SH, CC, DC, SE, HH, SY, SD analysed the data. VF, SH, CC, DC accessed and verified the data. VF, SH, CC, DC, SE, D’OC and JS wrote the manuscript. All authors contributed to data interpretation, reviewed, revised, and approved the manuscript, and accept responsibility to submit for publication.

## Competing interests

Prof Karen Canfell reports that she is co-PI of an investigator-initiated trial of cervical screening, "Compass", run by the Australian Centre for Prevention of Cervical Cancer (ACPCC), which is a government-funded not-for-profit charity. Compass receives infrastructure support from the Australian government and the ACPCC has received equipment and a funding contribution from Roche Molecular Diagnostics, USA. KC is also co-PI on a major implementation program *Elimination of Cervical Cancer in the Western Pacific* which has received support from the Minderoo Foundation and the Frazer Family Foundation and equipment donations from Cepheid Inc.”.

Dr Michael Caruana is also an investigatoe on Compass. However, neither KC nor MC, not their institution, have received direct funding from commercial organisations.

Other authors declare no conflict of interest.

## Data Availability

All the original data for this study are available upon reasonable request to the corresponding authors (KC or JS).
