# Supplementary material for: Are patients with cancer at higher risk of COVID-19-related death? A systematic review and critical appraisal of the early evidence
Source: J Cancer Policy. 2022 Sep;33:None. doi: 10.1016/j.jcpo.2022.100340 (PMC9169424; doi:10.1016/j.jcpo.2022.100340)
Supplement: Supplementary file 1 — Supplementary material [file mmc1.docx]

**Do COVID-19 patients with cancer have a higher risk of COVID-19-related death than those without cancer? A systematic review and critical appraisal of the early evidence.**

**Supplementary materials**

**Contents**

[**Supplementary Methods** 2](#_Toc86233405)

[**Supplementary Figures** 3](#_Toc86233406)

[Supplementary Figure 1 3](#_Toc86233407)

[Supplementary Figure 2 4](#_Toc86233408)

[Supplementary Figure 3 5](#_Toc86233409)

[Supplementary Figure 4 6](#_Toc86233410)

[Supplementary Figure 5 7](#_Toc86233411)

[Supplementary Figure 6 8](#_Toc86233412)

[Supplementary Figure 7 9](#_Toc86233413)

[Supplementary Figure 8 10](#_Toc86233414)

[Supplementary Figure 9 11](#_Toc86233415)

[Supplementary Figure 10 12](#_Toc86233416)

[Supplementary Figure 11 13](#_Toc86233417)

[Supplementary Figure 12 14](#_Toc86233418)

[Supplementary Figure 13 15](#_Toc86233419)

[Supplementary Figure 14 16](#_Toc86233420)

[Supplementary Figure 15 17](#_Toc86233421)

[Supplementary Figure 16 18](#_Toc86233422)

[Supplementary Figure 17 19](#_Toc86233423)

[Supplementary Figure 18 20](#_Toc86233424)

[**Supplementary Tables** 21](#_Toc86233425)

[Supplementary Table 1 21](#_Toc86233426)

[Supplementary Table 2 22](#_Toc86233427)

[Supplementary Table 3 24](#_Toc86233428)

[Supplementary Table 4 26](#_Toc86233429)

[Supplementary Table 5 36](#_Toc86233430)

[Supplementary Table 6 39](#_Toc86233431)

## **Supplementary Methods**

**Additional information on assessment of heterogeneity**

The χ^2^ test statistic assesses the amount of variation in a set of estimates and small p-values for the χ^2^ statistic imply that there is more heterogeneity present than would be expected by chance. However, the χ^2^test is not a particularly sensitive test and lack of statistical significance does not necessarily mean there is no heterogeneity. Consequently, to assess heterogeneity we also used the I^2^ statistic which corresponds to the proportion of variation between estimates that is due to heterogeneity rather than chance.[[1](#_ENREF_1)]

**Additional information on the assessment of the overlap in people included in different studies**

For the 96 studies that satisfied our inclusion criteria, we specifically assessed overlap in data included in studies that were pooled in each meta-analysis. However, there were overlapping patient groups included in different analyses. To estimate the total number of patients included in at least one analysis, we identified groups of studies that were non-independent, and only counted the number of patients from the largest study in each group. This yielded a conservative estimate of the total number of patients.

As the main focus in the results was on studies that reported adjusted effect estimates, we also specifically considered overlaps in the data used for the 18 studies that reported effect estimates adjusted for at least age. We identified several studies that were non-independent: 1) Tian et al.[[2](#_ENREF_2)] and Xie et al.[[3](#_ENREF_3)], and 2) the COVIDSurg Collaboration[[4](#_ENREF_4)] report and several other studies. We were unable to assess the possible overlap in patient groups reported by Docherty et al.[[5](#_ENREF_5)] and Galloway et al.[[6](#_ENREF_6)] Therefore, conservatively, we assessed that the 18 studies covered at least 15 independent datasets, all of which reported effect estimates indicating a positive association between pre-existing cancer and death after developing COVID-19.

## **Supplementary Figures**

Supplementary Figure 1 **(Analysis 1 in Table 2): Unadjusted odds ratio for COVID-19-related death, for people with pre-existing diagnosis of any cancer, compared to those without.**

Supplementary Figure 2 **(Analysis 1 in Table 2 grouped by country): Unadjusted odds ratio for COVID-19-related death, for people with pre-existing diagnosis of any cancer, compared to those without.**

Supplementary Figure 3 **(Analysis 1 in Table 2 grouped by source of population): Unadjusted odds ratio for COVID-19-related death, for people with pre-existing diagnosis of any cancer, compared to those without.**

Supplementary Figure 4 **(Analysis 1 in Table 2 grouped by publication status): Unadjusted odds ratio for COVID-19-related death, for people with pre-existing diagnosis of any cancer, compared to those without.**

Supplementary Figure 5 **(Analysis 3 in Table 2): Unadjusted hazard ratio for COVID-19-related death, for people with pre-existing diagnosis of any cancer, compared to those without.**

Supplementary Figure 6 **(Analysis 2 in Table 2 grouped by country): Adjusted odds/risk ratio for COVID-19-related death, for people with pre-existing diagnosis of any cancer, compared to those without.**

Supplementary Figure 7 **(Analysis 2 in Table 2 grouped by source of population): Adjusted odds/risk ratio for COVID-19-related death, for people with pre-existing diagnosis of any cancer, compared to those without.**

Supplementary Figure 8 **(Analysis 2 in Table 2 grouped by adjusted covariates): Adjusted odds/risk ratio for COVID-19-related death, for people with pre-existing diagnosis of any cancer, compared to those without.**

Supplementary Figure 9 **(Analysis 4 in Table 2): Adjusted hazard ratio for COVID-19-related death, for people with pre-existing diagnosis of any cancer, compared to those without.**

Supplementary Figure 10 **(Analysis 6 in Table 2): Unadjusted rate ratio for COVID-19-related death, for people with pre-existing diagnosis of any cancer, compared to people with COVID-19 diagnosis.**

Supplementary Figure 11 **(Analysis 7 in Table 2): Unadjusted odds ratio for COVID-19-related death, for people with active cancer, compared to people without previous diagnosis of cancer.**

Supplementary Figure 12 **(Analysis 8 in Table 2): Unadjusted odds ratio for COVID-19-related death, for people with active cancer, compared to people without active cancer.**

Supplementary Figure 13 **(Analysis 11 in Table 2): Adjusted hazard ratio for COVID-19-related death, for people with active cancer, compared to people without active cancer.**

Supplementary Figure 14 **(Analysis 13 in Table 2): Unadjusted hazard ratio for COVID-19-related death, for people with a pre-existing diagnosis of haematological cancer, compared to people without pre-existing diagnosis of cancer.**

Supplementary Figure 15 **(Analysis 14 in Table 2): Unadjusted rate ratio for COVID-19-related death, for people with pre-existing diagnosis of haematological cancer, compared to people with COVID-19 diagnosis.**

Supplementary Figure 16 **(Analysis 16 in Table 2): Unadjusted odds ratio for COVID-19-related death, for people with pre-existing lung cancer diagnosis, compared to people without pre-existing cancer diagnosis.**

Supplementary Figure 17 **(Analysis 1 in Table 2 using fixed-effect rather than random-effects): Unadjusted odds ratio for COVID-19-related death, for people with pre-existing diagnosis of any cancer, compared to those without.**

Supplementary Figure 18 **(Analysis 2 in Table 2 using fixed-effect rather than random-effects): Adjusted odds/risk ratio for COVID-19-related death, for people with pre-existing diagnosis of any cancer, compared to those without.**

## **Supplementary Tables**

Supplementary Table 1**: Search strategy used to identify potentially relevant records in Medline and Embase databases**

| **#** | **Searches (Embase Classic+Embase 1947 to 2020 July 01, Ovid MEDLINE(R) ALL 1946 to July 01, 2020)** | **Results** |
| --- | --- | --- |
| 1 | ("2019 nCoV" or 2019nCoV or "2019 novel coronavirus" or ((coronavirus or "corona virus") and (Huanan or Hubei or Wuhan)) or "coronavirus 19" or "coronavirus disease 19" or "coronavirus disease-2019" or "COVID 19" or COVID19 or "nCov 2019" or "new coronavirus" or "new coronaviruses" or "novel coronavirus" or "novel coronaviruses" or "novel corona virus" or "SARS CoV2" or "SARS CoV 2" or SARSCoV2 or "SARSCoV 2" or "SARS-coronavirus-2" or "SARS-like coronavirus" or "Severe Acute Respiratory Syndrome Coronavirus 2").tw. | 53972 |
| 2 | (cancer* or oncolog* or malignan* or tumor* or tumour* or carcinoma* or co-morbidit* or comorbidit* or multi-morbidit* or multimorbidit*).mp. | 9763266 |
| 3 | 1 and 2 | 5000 |
| 4 | limit 3 to yr="2020 -Current" | 4929 |
| 5 | remove duplicates from 4 | 3097 |

Supplementary Table 2**: Risk of bias assessment tool for cohort studies**

| **Risk of bias assessment tool for observational cohort studies (comprising 2018 adapted Newcastle-Ottawa Scale[**[**7**](#_ENREF_7)**], McMaster university tool[**[**8**](#_ENREF_8)**] and ROBINS-I tool[**[**9**](#_ENREF_9)**])**  **1. Exposed and comparison (unexposed) populations and selection of cohort(s)**   1. Drawn from the same population **(low risk)** 2. Drawn from different populations but unlikely to introduce bias **(moderate risk)** 3. Drawn from different populations and likely to introduce bias OR insufficient information to tell **(high risk)**   **2. Were co-interventions similar between groups?**  *(Could the difference in outcome for exposed and unexposed be due to differences in COVID or other management and not cancer?)*   1. Most or all co-interventions similar between exposed and unexposed groups OR some or all co-interventions different between exposed and unexposed but unlikely to introduce bias (i.e. irrelevant) **(low risk)** 2. Some or all co-interventions different between exposed and unexposed groups and likely to introduce bias OR insufficient information to tell **(high risk)**   **3. Nature and measurement of exposure**  *(Is exposure measured blind to outcome? Addresses blinding and reliability of exposure ascertainment)*   1. Objective measurements from pre-existing records or baseline physical or biological assessment blind to outcome status **(low risk)** 2. Objective measurements from pre-existing records or baseline physical or biological assessment not blind or not known to be blind to outcome status OR structured interview **(moderate risk)** 3. Self-administered questionnaire OR insufficient information to tell **(high risk)**   **4. Was outcome of interest present at the time to which the exposure measurement refers?**  *(Asks was the outcome likely to alter the exposure measured)*   1. No **(low risk)** 2. Yes but outcome unlikely to affect exposure measurement **(moderate risk)** 3. Yes and outcome likely to affect exposure measurement OR insufficient information to tell **(high risk)**   **5. Nature and measurement of outcome**  *(Was assignment of the outcome influenced by knowledge of the exposure?)*   1. Objective outcome measurement unlikely to be influenced by knowledge of exposure **(low risk)** 2. Objective outcome measurement possibly influenced by knowledge of exposure **(moderate risk)** 3. Objective outcome measurement probably influenced by knowledge of exposure OR self-reported outcome OR insufficient information to tell **(high risk)**   **6. Completeness of follow up**  *(What % of original sample was followed up for ascertainment of outcome?)*   1. Active or passive follow-up of participants with methods of ascertainment of outcome and death clearly described AND with methods of ascertainment of emigration from population-at-risk clearly described or censoring at date of last follow-up OR there is a plausible estimate of >90% follow-up **(low risk)** 2. Active or passive follow-up with methods of ascertainment of outcome, death and emigration from population-at-risk not clearly described OR there is a plausible estimate of 70 – 90% follow-up **(moderate risk)** 3. Active or passive follow-up with methods of ascertainment of one or more of outcome, death or emigration not described OR there was probably <70% follow-up OR insufficient information to tell **(high risk)**   **7. Differences in follow up**  *(Did the methods of follow-up or sources of information and the proportions followed up differ between those with and without cancer?)*   1. Follow-up methods are the same and likely to achieve the same completeness of follow-up for exposed and unexposed participants **(low risk)** 2. Completeness of follow-up for exposed and unexposed participants is unlikely to be the same but difference between the two is, or would be likely to be, small (<10%) **(moderate risk)** 3. Completeness of follow-up for exposed and unexposed participants is very unlikely to be the same and difference between the two is, or is likely to be, large (>10%) OR insufficient information to tell **(high risk)**   **8. Exclusions due to missing data on any variables**  *(Have any patients been excluded from the study or analyses due to missing data (including cancer status or comorbidity data))*   1. Data for <10% of patients excluded due to missing data or explicitly state data for all patients identified presented **(low risk)** 2. Data for 10-30% of patients excluded due to missing data and difference between % exposed and % unexposed excluded due to missing data <10% **(moderate risk)** 3. Data for >30% patients excluded due to missing data, the difference between % exposed and % unexposed excluded due to missing data >10% or exclusions due to missing data not reported **(high risk)**   **9. Control of confounding**  *a. Comparability of exposed and unexposed cohorts with respect to potentially important confounding variables*   1. Age and other potentially important confounders measured and controlled by design or in analysis **(low risk)** 2. Age and some but not all other potentially important confounders controlled by design or in analysis **(moderate risk)** 3. No potentially important confounders or only age controlled by design or in analysis OR insufficient information to tell **(high risk)**   *b. Can we be confident in the assessment of the presence or absence of prognostic factors?*  *(How reliable are all the covariates included in analysis (not just age and sex)?)*   1. Yes **(low risk)** 2. No or insufficient information to tell **(high risk)** 3. No adjustments made or only adjusted for age and/or sex **(not applicable)**   *c. Covariates are appropriately included in the analysis*   1. NO variable measuring the same underlying concept or lying in the same causal pathway as the exposure variable under study IS included as a covariate in the statistical analysis models **(low risk)** 2. One or more variables that **could** both reflect comorbidity status (unclear as to whether confounder) ***and be*** on the same causal pathway as the exposure variable under study is included as a covariate in statistical analysis models **(moderate risk)** 3. ONE OR MORE variables measuring the same underlying concept or lying in the same causal pathway as the exposure variable under study IS included as a covariate in statistical analysis models OR insufficient information to tell **(high risk)** 4. No adjustments made or only adjusted for age and/or sex **(not applicable)**   **Key to overall risk of bias rating:**  **High risk of bias –** high risk of bias for any domain  **Moderate risk of bias –** moderate or low risk of bias for all domains, no high-risk domains  **Low risk of bias –** all domains low risk of bias, no moderate or high-risk domains |
| --- |

Supplementary Table 3**: Risk of bias assessment tool for nested case-control studies**

| **Risk of Bias assessment tool for nested case-control studies (comprising 2015 adapted Newcastle-Ottawa Scale[**[**10**](#_ENREF_10)**], McMaster university tool[**[**8**](#_ENREF_8)**] and ROBINS-I tool[**[**9**](#_ENREF_9)**])**  **Bias in selection of participants into nested case-control study**   1. *Sources of cases (deaths) and controls (survivors)* 2. Drawn from the same population* (**low risk**) 3. Drawn from different populations but unlikely to introduce bias (**moderate risk**) 4. Drawn from different populations and likely to introduce bias OR insufficient information to tell (**high risk**)   * This will usually be the case when a case-control study is nested in a single cohort containing exposed and unexposed people and cases accrue during follow-up of the whole cohort.   1. *Selection of cases and controls* 2. Cases and controls are randomly selected from all available cases and controls; controls matched to cases by risk set* (either at selection or during analysis) (**low risk**) 3. Only one of the two criteria in 1 is met (**moderate risk**) 4. Neither criterion in 1 is met OR insufficient information to tell (**high risk**)   *Risk set defined by sex, age group, date of entry into cohort and date of case-defining event  **Bias due to error in outcome measurement**   1. *Definition of cases (outcome)* 2. Outcome precisely specified and with pathological or other objective confirmation (**low risk**) 3. Outcome precisely specified but without known pathological or other objective confirmation OR outcome precisely specified, self-reported and cases blind to hypotheses related to outcome (**moderate risk**) 4. Outcome imprecisely specified OR outcome self-reported and cases not blind to hypotheses related to outcome OR insufficient information to tell (**high risk**) 5. *Definition of controls* 6. Objective evidence of no past history of outcome of interest (**low risk**) 7. Self-report of no past history of outcome of interest OR insufficient information to tell (**moderate risk**) 8. *Was outcome of interest likely to have been absent at the time to which the exposure refers?* 9. Yes (**low risk**) 10. No but outcome unlikely to affect exposure measurement (**moderate risk**) 11. No and outcome likely to affect exposure measurement OR insufficient information to tell (**high risk**)   **Bias due to error in exposure measurement**   1. *Measurement of exposure (pre-existing cancer)* 2. Objective measurements from pre-existing records or baseline* physical or biological assessment or structured interview, each blind to case or control status (**low risk**) 3. Objective measurements from pre-existing records or baseline* physical or biological assessment not blind to case or control status OR structured interview blind to case or control status (**moderate risk**) 4. Structured interview not blind to case or control status OR self-administered questionnaire OR insufficient information to tell (**high risk**)   * Existing at or before baseline, where baseline is the time at which a participant is recorded to have entered the cohort or, if obtained after baseline, a time before onset of symptoms of the outcome or any likely effect of the developing outcome on the exposure   1. *Was the same method used to measure exposure (cancer) in cases and controls?* 2. Yes (**low risk**) 3. No OR insufficient information to tell (**high risk**)   **Bias due to missing data**   1. *Completeness of follow-up of cohort* 2. Active or passive follow-up of participants with methods for ascertainment of outcome and death clearly described AND with methods for ascertainment of emigration from population-at-risk clearly described or censoring at date of last follow-up OR there is a plausible estimate of >90% follow-up (**low risk**) 3. Active or passive follow-up with methods for ascertainment of outcome, death and emigration from population-at-risk not clearly described OR there is a plausible estimate of 70-90% follow-up (**moderate risk**) 4. Active or passive follow-up with methods for ascertainment of one or more of outcome, death or emigration not described OR there was probably <70% follow-up OR insufficient information to tell (**high risk**) 5. *Difference in follow-up between exposed and non-exposed members of cohort* 6. Follow-up methods are the same and likely to achieve the same completeness of follow-up in exposed and unexposed participants (**low risk**) 7. Completeness of follow-up in exposed and unexposed participants is unlikely to be the same but difference between the two is, or would be likely to be, small (<10%) (**moderate risk**) 8. Completeness of follow-up in exposed and unexposed participants is very unlikely to be the same and difference between the two is, or is likely to be, large (>10%) OR insufficient information to tell (**high risk**)      1. *Exclusions due to missing data on other variables* (Are all patients included in the analyses?) 2. Data for < 10% of patients excluded due to missing data or explicitly state data for all patients identified presented (**low risk**) 3. Data for 10-30% of patients excluded due to missing data and difference between % exposed and % unexposed excluded due to missing data <10% (**moderate risk**) 4. Data for >30% patients excluded due to missing data, the difference between % exposed and % unexposed excluded due to missing data > 10% or exclusions due to missing data not reported (**high risk**) 5. No adjusted analyses presented (**not applicable**)   **Bias due to confounding**   1. *Comparability of cases and controls with respect to potentially important confounding variables* 2. Age and other potentially important confounders measured and controlled by design or in analysis (**low risk**) 3. Age and sex (both essential) controlled by design or in analysis (**moderate risk**) 4. Age or sex (neither or only one) controlled by design or in analysis OR insufficient information to tell (**high risk**) 5. *Were co-interventions similar between groups?* (Could the difference in outcome for exposed and unexposed be due to differences in COVID or other management and not cancer?) 6. Most or all co-interventions similar between exposed and unexposed groups OR some or all co-interventions different between exposed and unexposed but unlikely to introduce bias (i.e. irrelevant) (**low risk**) 7. Some or all co-interventions different between exposed and unexposed groups and likely to introduce bias OR insufficient information to tell (**high risk**) 8. *Assessment of the presence or absence of prognostic factors* (How reliable are all the covariates adjusted for (not just age and sex)?) 9. Yes (**low risk**) 10. No or insufficient information to tell (**high risk**) 11. No adjustments made or if only adjusted for age and/or sex (**not applicable**) 12. *Covariates are appropriately included in statistical analysis models* 13. Variables measuring the same underlying concept or lying in the same causal pathway ARE NOT included together as covariates in statistical analysis models (**low risk**) 14. Variables measuring the same underlying concept or lying in the same causal pathway ARE included together as covariates in statistical analysis models OR insufficient information to tell (**high risk**) 15. No adjustments made OR If only adjusted for age and/or sex (**not applicable**)   **Analysis bias**   1. *Analysis appropriate to design* 2. When controls are frequency matched to cases, matching variables are controlled in the analysis OR when controls are individually matched to cases, a conditional analysis is used or matching variables are controlled in the analysis (**low risk**) 3. None of the above OR insufficient information to tell (**high risk**) 4. Controls are not matched in any way (**not applicable**)   **Key to overall risk of bias rating:**  **High risk of bias** – high risk of bias for any domain  **Moderate risk of bias** – moderate or low risk of bias for all domains, no high risk domains  **Low risk of bias** – all domains low risk of bias, no moderate or high risk domains |
| --- |

Supplementary Table 4**: List of 380 studies excluded from review at full text screening stage, and reason(s) for exclusion**

| **Study author** | **Available from** | **Reason(s) for exclusion** |
| --- | --- | --- |
| Aaroe, A., et al. | https://dx.doi.org/10.1093/neuonc/noaa096 | Excluded publication type or study design, or letter or comment with no relevant primary data |
| Adams, M., et al. | https://doi.org/10.1101/2020.05.02.20088781 | No population of interest |
| Adjei Boakye, E., et al. | https://dx.doi.org/10.1002/hed.26170 | Excluded publication type or study design, or letter or comment with no relevant primary data |
| Afshar, Z. M., et al. | https://dx.doi.org/10.1016/j.jinf.2020.05.062 | Excluded publication type or study design, or letter or comment with no relevant primary data, No population of interest |
| Alkhathami, M. G., et al. | <https://doi.org/10.1101/2020.06.01.20119271> | Excluded publication type or study design, or letter or comment with no relevant primary data |
| Almazeedi, S., et al. | <https://doi.org/10.1101/2020.05.09.20096495> | Excluded publication type or study design, or letter or comment with no relevant primary data, No comparator of interest |
| Alsofayan, Y. M., et al. | https://dx.doi.org/10.1016/j.jiph.2020.05.026 | Excluded publication type or study design, or letter or comment with no relevant primary data, No comparator of interest |
| Andre, N., et al. | https://dx.doi.org/10.1002/pbc.28392 | Excluded publication type or study design, or letter or comment with no relevant primary data |
| Arentz, M., et al. | http://dx.doi.org/10.1001/jama.2020.4326 | No outcome of interest, No exposure of interest |
| Argenziano, M. G., et al. | https://doi.org/10.1101/2020.04.20.20072116 | No outcome of interest |
| Asokan, I., et al. | https://dx.doi.org/10.1007/s11912-020-00945-4 | Excluded publication type or study design, or letter or comment with no relevant primary data |
| Assaad, S., et al. | https://dx.doi.org/10.1016/j.ejca.2020.05.028 | Excluded publication type or study design, or letter or comment with no relevant primary data, No population of interest |
| Bajwa, S. J. S., et al. | http://dx.doi.org/10.4103/ija.IJA_573_20 | Excluded publication type or study design, or letter or comment with no relevant primary data |
| Barrasa, H., et al. | https://dx.doi.org/10.1016/j.accpm.2020.04.001 | No population of interest, No exposure of interest |
| Bean, D. M., et al. | https://dx.doi.org/10.1002/ejhf.1924 | No population of interest, No exposure of interest |
| Becchetti, C., et al. | https://dx.doi.org/10.1136/gutjnl-2020-321923 | No population of interest |
| Bello-Chavolla, O. Y., et al. | https://dx.doi.org/10.1093/gerona/glaa163 | No exposure of interest |
| Bellou, V., et al. | https://doi.org/10.1101/2020.05.13.20100495 | Excluded publication type or study design, or letter or comment with no relevant primary data |
| Best, J., et al. | https://dx.doi.org/10.1016/j.clon.2020.05.021 | Excluded publication type or study design, or letter or comment with no relevant primary data |
| Biagi, A., et al. | https://dx.doi.org/10.1002/jmv.26147 | No exposure of interest, No population of interest |
| Biagioli, V., et al. | https://dx.doi.org/10.1016/j.ejca.2020.05.022 | Excluded publication type or study design, or letter or comment with no relevant primary data |
| Blandino, G., et al. | https://dx.doi.org/10.1186/s13046-020-01575-1 | Excluded publication type or study design, or letter or comment with no relevant primary data |
| Boddington, N. L., et al. | https://doi.org/10.1101/2020.05.18.20086157 | No outcome of interest, No population of interest |
| Borobia, A. M., et al. | https://doi.org/10.1101/2020.04.29.20080853 | Preprint subsequently published |
| Boulad, F., et al. | https://dx.doi.org/10.1001/jamaoncol.2020.2028 | No exposure of interest, No population of interest |
| Bravi, F., et al. | <https://doi.org/10.1101/2020.05.21.20109082> | Preprint subsequently published |
| Bravi, F., et al. | https://doi.org/10.1101/2020.05.21.20109082 | No outcome of interest |
| Brill, S. E., et al. | https://dx.doi.org/10.1186/s12916-020-01665-z | No outcome of interest, No exposure of interest |
| Brookman-May, S. D., et al. | https://dx.doi.org/10.1016/j.eururo.2020.06.014 | Excluded publication type or study design, or letter or comment with no relevant primary data |
| Brunetti, O., et al. | https://dx.doi.org/10.3389/fonc.2020.00734 | Excluded publication type or study design, or letter or comment with no relevant primary data |
| Buckner, F. S., et al. | https://dx.doi.org/10.1093/cid/ciaa632 | No outcome of interest |
| Buenen, A. G., et al. | https://pubmed.ncbi.nlm.nih.gov/32392009/ | Non-English language |
| Burki, T. K. | https://dx.doi.org/10.1016/S1470-2045(20)30201-1 | Excluded publication type or study design, or letter or comment with no relevant primary data |
| Burn, E., et al. | https://dx.doi.org/10.1101/2020.04.22.20074336 | No outcome of interest |
| Busetto, L., et al. | https://dx.doi.org/10.1002/oby.22918 | No outcome of interest |
| Caffo, O., et al. | https://dx.doi.org/10.1016/j.annonc.2020.06.005 | Excluded publication type or study design, or letter or comment with no relevant primary data |
| Cai, C., et al. | https://dx.doi.org/10.1016/j.jinf.2020.05.028 | Excluded publication type or study design, or letter or comment with no relevant primary data |
| Cai, Q., et al. | https://dx.doi.org/10.2337/dc20-0576 | No outcome of interest |
| Cao, M., et al. | https://dx.doi.org/10.1101/2020.03.04.20030395 | No outcome of interest |
| Caramelo F., et al. | <https://www.medrxiv.org/content/10.1101/2020.02.24.20027268v1> | No outcome of interest |
| Carta, M. G., et al. | https://dx.doi.org/10.26355/eurrev_202004_20794 | Excluded publication type or study design, or letter or comment with no relevant primary data |
| CDC Covid-19 Response Team | https://dx.doi.org/10.15585/mmwr.mm6913e2 | No outcome of interest, No exposure of interest |
| Cen, Y., et al. | https://dx.doi.org/10.1016/j.cmi.2020.05.041 | No exposure of interest, No outcome of interest |
| Chang T., et al. | https://doi.org/10.1101/2020.07.03.20145581 | No exposure of interest |
| Chao, J. Y., et al. | https://dx.doi.org/10.1016/j.jpeds.2020.05.006 | No outcome of interest, No population of interest |
| Chen, G., et al. | http://dx.doi.org/10.1172/JCI137244 | No exposure of interest |
| Chen, J., et al. | <https://doi.org/10.1016/j.jinf.2020.03.004> | Excluded publication type or study design, or letter or comment with no relevant primary data |
| Chen, P., et al. | https://dx.doi.org/10.12659/MSM.923985 | No exposure of interest |
| Chen, Q., et al. | https://dx.doi.org/10.1007/s15010-020-01432-5 | No outcome of interest |
| Chen, R., et al. | <https://doi.org/10.1016/j.chest.2020.04.010> | No outcome of interest |
| Chen, W., et al. | <https://papers.ssrn.com/sol3/papers.cfm?abstract_id=3559599> | Excluded publication type or study design, or letter or comment with no relevant primary data |
| Cherri, S., et al. | https://dx.doi.org/10.1016/j.mehy.2020.109758 | Excluded publication type or study design, or letter or comment with no relevant primary data |
| Christensen, D. M., et al. | https://dx.doi.org/10.1007/s11606-020-05991-z | No outcome of interest |
| Chudasama, Y. V., et al. | https://dx.doi.org/10.1016/j.dsx.2020.06.003 | Excluded publication type or study design, or letter or comment with no relevant primary data, No outcome of interest |
| Climent, F. J., et al. | https://dx.doi.org/10.1016/j.rec.2020.04.011 | Excluded publication type or study design, or letter or comment with no relevant primary data |
| Cook, G., et al. | https://dx.doi.org/10.1111/bjh.16874 | Excluded publication type or study design, or letter or comment with no relevant primary data |
| COVID-19 National Incident Room Surveillance Team | https://dx.doi.org/10.33321/cdi.2020.44.43 | No exposure of interest |
| COVID-19 National Incident Room Surveillance Team | https://dx.doi.org/10.33321/cdi.2020.44.45 | No exposure of interest, No outcome of interest |
| COVID-19 National Incident Room Surveillance Team | https://dx.doi.org/10.33321/cdi.2020.44.51 | No exposure of interest |
| COVID-19 National Incident Room Surveillance Team | https://dx.doi.org/10.33321/cdi.2020.44.52 | No exposure of interest |
| COVID-19 National Incident Room Surveillance Team | https://dx.doi.org/10.33321/cdi.2020.44.54 | No exposure of interest, No outcome of interest |
| da Fonseca, L., et al. | https://dx.doi.org/10.1111/liv.14545 | Excluded publication type or study design, or letter or comment with no relevant primary data |
| Dagan, N., et al. | <https://doi.org/10.1101/2020.05.20.20108571> | No outcome of interest |
| de Rojas, T., et al. | https://dx.doi.org/10.1002/pbc.28397 | Excluded publication type or study design, or letter or comment with no relevant primary data |
| Desai, A., et al. | <https://doi.org/10.1200/go.20.00097> | Excluded publication type or study design, or letter or comment with no relevant primary data |
| Di Giacomo, A. M., et al. | https://dx.doi.org/10.1200/GO.20.00097 | Excluded publication type or study design, or letter or comment with no relevant primary data |
| Di Lorenzo, G., et al. | https://dx.doi.org/10.1016/j.ejca.2020.04.026 | Excluded publication type or study design, or letter or comment with no relevant primary data |
| Ding, Y. Y., et al. | http://dx.doi.org/10.3389/fmed.2020.00140 | Excluded publication type or study design, or letter or comment with no relevant primary data |
| Docherty, A. B., et al. | https://doi.org/10.1101/2020.04.23.20076042 | Preprint subsequently published |
| Doglietto, F., et al. | https://dx.doi.org/10.1001/jamasurg.2020.2713 | No outcome of interest |
| Dorjee, K., et al. | https://doi.org/10.1101/2020.06.19.20135483 | Excluded publication type or study design, or letter or comment with no relevant primary data |
| Du, Y., et al. | https://dx.doi.org/10.1164/rccm.202003-0543OC | Excluded publication type or study design, or letter or comment with no relevant primary data, No population of interest |
| Duanmu, Y., et al. | https://dx.doi.org/10.1111/acem.14003 | No outcome of interest |
| Dufour, I., et al. | http://dx.doi.org/10.1007/s00277-020-04147-7 | Excluded publication type or study design, or letter or comment with no relevant primary data |
| Ebinger , J. E., et al. | https://doi.org/10.1101/2020.04.29.20084533 | No exposure of interest |
| Emami, A., et al. | <https://pubmed.ncbi.nlm.nih.gov/32232218/> | Excluded publication type or study design, or letter or comment with no relevant primary data |
| Escalera-Antezana, J. P., et al. | <https://pubmed.ncbi.nlm.nih.gov/32487789/> | No exposure of interest |
| Espinosa, O. A., et al. | https://dx.doi.org/10.1590/S1678-9946202062043 | Excluded publication type or study design, or letter or comment with no relevant primary data |
| Extance, A. | https://dx.doi.org/10.1136/bmj.m1174 | Excluded publication type or study design, or letter or comment with no relevant primary data |
| Fattizzo, B., et al. | <https://doi.org/10.1038/s41375-020-0877-y> | Excluded publication type or study design, or letter or comment with no relevant primary data, No outcome of interest |
| Feng, X., et al. | <https://doi.org/10.1101/2020.04.24.20078063> | No outcome of interest |
| Feng, Y., et al. | https://dx.doi.org/10.1164/rccm.202002-0445OC | No outcome of interest |
| Fong, D., et al. | <https://doi.org/10.1136/esmoopen-2020-000810> | Excluded publication type or study design, or letter or comment with no relevant primary data |
| Fratino, L., et al. | https://dx.doi.org/10.3389/fonc.2020.00648 | Excluded publication type or study design, or letter or comment with no relevant primary data, No population of interest |
| Fu, L., et al. | https://dx.doi.org/10.1016/j.jinf.2020.03.041 | Excluded publication type or study design, or letter or comment with no relevant primary data |
| Gampel, B., et al. | https://dx.doi.org/10.1002/pbc.28420 | No population of interest |
| Gao, Y., et al. | <https://doi.org/10.1101/2020.05.01.20087031> | Excluded publication type or study design, or letter or comment with no relevant primary data |
| Garassino, M. C., et al. | https://dx.doi.org/10.1016/S1470-2045(20)30314-4 | Excluded publication type or study design, or letter or comment with no relevant primary data |
| Garazzino, S., et al. | https://dx.doi.org/10.2807/1560-7917.ES.2020.25.18.2000600 | No population of interest |
| Giannakoulis, V. G., et al. | https://dx.doi.org/10.1200/GO.20.00225 | Excluded publication type or study design, or letter or comment with no relevant primary data |
| Gidari, A., et al. | https://dx.doi.org/10.1080/23744235.2020.1784457 | No exposure of interest |
| Gold, J. A. W., et al. | https://dx.doi.org/10.15585/mmwr.mm6918e1 | No outcome of interest |
| Gold, M. S., et al. | https://dx.doi.org/10.1080/00325481.2020.1786964 | Excluded publication type or study design, or letter or comment with no relevant primary data |
| Goyal P., et al. | <https://doi.org/10.1056/nejmc2010419> | No outcome of interest |
| Grasselli, G., et al. | https://dx.doi.org/10.1001/jama.2020.5394 | No outcome of interest |
| Guan, W. J., et al. | https://doi.org/10.1016%2Fj.jemermed.2020.04.004 | No outcome of interest |
| Guo, T., et al. | https://dx.doi.org/10.1159/000508734 | No exposure of interest |
| Guo, W., et al. | https://dx.doi.org/10.1002/dmrr.3319 | No outcome of interest |
| Han, P., et al. | <https://doi.org/10.2139/ssrn.3566133> | Excluded publication type or study design, or letter or comment with no relevant primary data |
| Harrison, E., et al. | <https://papers.ssrn.com/sol3/papers.cfm?abstract_id=3618215> | Excluded publication type or study design, or letter or comment with no relevant primary data |
| Hatzl, S., et al. | https://dx.doi.org/10.1038/s41375-020-0914-x | No comparator of interest |
| He, B., et al. | https://dx.doi.org/10.1016/j.jinf.2020.05.069 | No exposure of interest |
| Helms, J., et al. | <https://doi.org/10.1007/s00134-020-06062-x> | No outcome of interest |
| Hernandez-Garduno, E. | https://dx.doi.org/10.1016/j.orcp.2020.06.001 | No exposure of interest |
| Ho, F. K., et al. | <https://doi.org/10.1101/2020.04.28.20083295> | No outcome of interest, No population of interest |
| Hochster, H. S. | <https://www.cancernetwork.com/view/oncology-time-covid-19> | Excluded publication type or study design, or letter or comment with no relevant primary data |
| Hong, K. S., et al. | http://dx.doi.org/10.3349/ymj.2020.61.5.431 | No outcome of interest |
| Hrusak, O., et al. | https://dx.doi.org/10.1016/j.ejca.2020.03.021 | No population of interest |
| Hu, K., et al. | https://dx.doi.org/10.3760/cma.j.cn121430-20200507-00364 | Non-English language, No exposure of interest |
| Hu, Y., et al. | https://dx.doi.org/10.1016/j.jcv.2020.104371 | Excluded publication type or study design, or letter or comment with no relevant primary data |
| Huang J., et al. | <https://doi.org/10.1002/jmv.25969> | No outcome of interest |
| Huang Y., et al | <https://doi.org/10.1101/2020.04.18.20070656> | No outcome of interest |
| Huang, C., et al. | https://dx.doi.org/10.1016/S0140-6736(20)30183-5 | No outcome of interest |
| Iaccarino, G., et al. | https://dx.doi.org/10.1161/HYPERTENSIONAHA.120.15324 | No exposure of interest |
| Ibrahim, L. F., et al. | https://dx.doi.org/10.1111/1742-6723.13550 | No population of interest |
| ICNARC | <https://www.icnarc.org/DataServices/Attachments/Download/c9b491af-ea80-ea11-9124-00505601089b> | No outcome of interest |
| Islam, M. S., et al. | <https://doi.org/10.1101/2020.05.23.20110965> | Excluded publication type or study design, or letter or comment with no relevant primary data |
| Israelsen, S. B., et al. | <https://pubmed.ncbi.nlm.nih.gov/32448405/> | No exposure of interest |
| Issitt. R. W., et al. | <https://doi.org/10.1101/2020.05.20.20107904> | No population of interest |
| Jain, V., et al. | https://dx.doi.org/10.1007/s00038-020-01390-7 | Excluded publication type or study design, or letter or comment with no relevant primary data |
| Jeon, J. et al. | <https://doi.org/10.1101/2020.05.17.20104729> | Excluded publication type or study design, or letter or comment with no relevant primary data |
| Ji, D., et al. | https://dx.doi.org/10.1093/cid/ciaa414 | No exposure of interest |
| Ji, W. et al. | <https://doi.org/10.1101/2020.05.08.20095174> | Preprint subsequently published, No outcome of interest |
| Ji, W., et al. | <https://doi.org/10.3346%2Fjkms.2020.35.e237> | No outcome of interest |
| Jin, J. M., et al. | https://dx.doi.org/10.3389/fpubh.2020.00152 | No exposure of interest |
| Joharatnam-Hogan, N., et al. | https://dx.doi.org/10.1186/s12916-020-01649-z | Excluded publication type or study design, or letter or comment with no relevant primary data |
| Jung, H. Y., et al. | https://dx.doi.org/10.3390/jcm9061688 | No population of interest |
| Jutzeler, C., et al. | <https://doi.org/10.1016/j.tmaid.2020.101825> | Excluded publication type or study design, or letter or comment with no relevant primary data |
| Kabarriti, R., et al. | http://dx.doi.org/10.1016/j.adro.2020.04.028 | Excluded publication type or study design, or letter or comment with no relevant primary data |
| Kahathuduwa, C., et al. | <http://dx.doi.org/10.2139/ssrn.3564410> | Excluded publication type or study design, or letter or comment with no relevant primary data |
| Kalinsky, K., et al. | https://dx.doi.org/10.1007/s10549-020-05667-6 | Excluded publication type or study design, or letter or comment with no relevant primary data |
| Kammar-Garcia, A., et al. | https://dx.doi.org/10.24875/RIC.20000207 | No exposure of interest |
| Kanellopoulos, A., et al. | http://dx.doi.org/10.1111/bjh.16856 | Excluded publication type or study design, or letter or comment with no relevant primary data |
| Kartik, A., et al. | https://dx.doi.org/10.1177/1010539520937103 | Excluded publication type or study design, or letter or comment with no relevant primary data, No population of interest |
| Kaspers, G. J. L. | https://dx.doi.org/10.1080/14737140.2020.1781621 | Excluded publication type or study design, or letter or comment with no relevant primary data |
| Kattan, J., et al. | https://dx.doi.org/10.2217/imt-2020-0077 | Excluded publication type or study design, or letter or comment with no relevant primary data |
| Kaushik, S., et al. | https://dx.doi.org/10.1016/j.jpeds.2020.06.045 | No population of interest |
| Kebisek, J., et al. | <https://pubmed.ncbi.nlm.nih.gov/32479103/> | No outcome of interest |
| Khamis, F., et al. | https://dx.doi.org/10.1016/j.jiph.2020.06.002 | No exposure of interest |
| Khan, M. A., et al. | <https://doi.org/10.1101/2020.05.08.20095968> | Excluded publication type or study design, or letter or comment with no relevant primary data |
| Kobayashi, Y., et al. | https://dx.doi.org/10.3802/jgo.2020.31.e65 | Excluded publication type or study design, or letter or comment with no relevant primary data |
| Kolin, D. A., et al. | <https://doi.org/10.1101/2020.05.05.20075507> | No outcome of interest |
| Kong, Q., et al. | https://dx.doi.org/10.1186/s12943-020-01209-2 | Excluded publication type or study design, or letter or comment with no relevant primary data |
| Kong, W., et al. | https://dx.doi.org/10.1016/j.tmaid.2020.101754 | No exposure of interest |
| Korean Society of Infectious Diseases., et al. | https://dx.doi.org/10.3346/jkms.2020.35.e132 | No population of interest |
| Kotecha, R. S. | https://dx.doi.org/10.1016/S1470-2045(20)30205-9 | Excluded publication type or study design, or letter or comment with no relevant primary data |
| Kuderer, N. M., et al. | https://dx.doi.org/10.1016/S0140-6736(20)31187-9 | Excluded publication type or study design, or letter or comment with no relevant primary data, No population of interest |
| Kuno, T., et al. | https://dx.doi.org/10.1016/j.ahj.2020.05.005 | No outcome of interest |
| Laccourreye, O., et al. | https://dx.doi.org/10.1016/j.anorl.2020.06.007 | No population of interest |
| Lagadinou, M., et al. | <https://pubmed.ncbi.nlm.nih.gov/32532944/> | No outcome of interest |
| Lagier, J. C., et al. | https://dx.doi.org/10.1016/j.tmaid.2020.101791 | No outcome of interest |
| Lai, P. H., et al. | https://dx.doi.org/10.1001/jamacardio.2020.2488 | Excluded publication type or study design, or letter or comment with no relevant primary data, No population of interest |
| Lee, K. A., et al. | <https://doi.org/10.1101/2020.05.20.20103762> | Excluded publication type or study design, or letter or comment with no relevant primary data, No population of interest |
| Lee, L. Y. W., et al. | https://dx.doi.org/10.1016/S0140-6736(20)31173-9 | Excluded publication type or study design, or letter or comment with no relevant primary data, No population of interest |
| Leonel, A.C.L.D.S., et al. | https://dx.doi.org/10.1016/j.oraloncology.2020.104739 | Excluded publication type or study design, or letter or comment with no relevant primary data |
| Leonetti, A., et al. | https://dx.doi.org/10.1016/j.ejca.2020.04.004 | Excluded publication type or study design, or letter or comment with no relevant primary data |
| Leung, C. | https://dx.doi.org/10.1002/rmv.2103 | Excluded publication type or study design, or letter or comment with no relevant primary data, No exposure of interest |
| Leung, C. | https://dx.doi.org/10.1016/j.mad.2020.111255 | No exposure of interest |
| Lewis, P. J., et al. | https://dx.doi.org/10.1016/j.clon.2020.05.007 | Excluded publication type or study design, or letter or comment with no relevant primary data |
| Li, B., et al. | https://dx.doi.org/10.1007/s00392-020-01626-9 | Excluded publication type or study design, or letter or comment with no relevant primary data, No exposure of interest |
| Li, J., et al. | <https://doi.org/10.1001/jamacardio.2020.1624> | No population of interest |
| Li, J., et al. | https://dx.doi.org/10.1016/j.virusres.2020.198043 | No outcome of interest |
| Li, J., et al. | https://dx.doi.org/10.2196/19636 | No exposure of interest |
| Li, J., et al. | https://dx.doi.org/10.1016/j.ajic.2020.06.008 | Excluded publication type or study design, or letter or comment with no relevant primary data |
| Li, K., et al. | https://dx.doi.org/10.1097/RLI.0000000000000672 | No exposure of interest |
| Li, W., et al. | <https://doi.org/10.1038/s41375-020-0853-6> | Excluded publication type or study design, or letter or comment with no relevant primary data, No population of interest |
| Li, X., et al. | http://dx.doi.org/10.1016/j.ijid.2020.03.053 | No outcome of interest, No population of interest |
| Li, X., et al. | http://dx.doi.org/10.1016/j.jaci.2020.04.006 | No outcome of interest |
| Li, Y., et al. | https://dx.doi.org/10.1038/s41374-020-0431-6 | No population of interest, No exposure of interest |
| Liang, W. H., et al. | https://dx.doi.org/10.1183/13993003.00562-2020 | No outcome of interest |
| Liang, W., et al. | https://dx.doi.org/10.1016/S1470-2045(20)30096-6 | No outcome of interest |
| Liang, W., et al. | https://dx.doi.org/10.1001/jamainternmed.2020.2033 | No outcome of interest |
| Liang, X., et al. | https://dx.doi.org/10.1016/j.clon.2020.03.016 | Excluded publication type or study design, or letter or comment with no relevant primary data |
| Lippi, G., et al. | http://dx.doi.org/10.1002/jmv.25860 | No comparator of interest, Excluded publication type or study design, or letter or comment with no relevant primary data |
| Lithander, F. E., et al. | https://dx.doi.org/10.1093/ageing/afaa093 | Excluded publication type or study design, or letter or comment with no relevant primary data |
| Liu, B. L., et al. | https://dx.doi.org/10.3760/cma.j.cn112152-20200221-00110 | Non-English language |
| Liu, K., et al. | https://dx.doi.org/10.1097/CM9.0000000000000744 | No outcome of interest |
| Liu, T., et al. | https://dx.doi.org/10.1002/ijc.33148 | No outcome of interest, Excluded publication type or study design, or letter or comment with no relevant primary data |
| Lokken, E. M., et al. | https://dx.doi.org/10.1016/j.ajog.2020.05.031 | No population of interest |
| Long, L., et al. | https://dx.doi.org/10.1183/13993003.00990-2020 | No exposure of interest |
| Lorente-Ros, A., et al. | https://dx.doi.org/10.5603/CJ.a2020.0089 | No outcome of interest |
| Louapre, C., et al. | https://dx.doi.org/10.1001/jamaneurol.2020.2581 | No population of interest |
| Louhaichi, S., et al. | <https://pubmed.ncbi.nlm.nih.gov/32395788/> | Non-English language |
| Lovell, N., et al. | https://dx.doi.org/10.1016/j.jpainsymman.2020.04.015 | No outcome of interest, No population of interest |
| Lu, X., et al. | https://dx.doi.org/10.1056/NEJMc2005073 | No population of interest |
| Ludvigsson, J. F. | http://dx.doi.org/10.1111/apa.15270 | Excluded publication type or study design, or letter or comment with no relevant primary data |
| Lui, T. K., et al. | https://dx.doi.org/10.1053/j.gastro.2020.05.037 | No outcome of interest, No population of interest |
| Luo, J., et al. | https://dx.doi.org/10.1016/j.annonc.2020.06.007 | Excluded publication type or study design, or letter or comment with no relevant primary data |
| Luo, J., et al. | https://dx.doi.org/10.1158/2159-8290.CD-20-0596 | Excluded publication type or study design, or letter or comment with no relevant primary data |
| Luo, M., et al. | http://dx.doi.org/10.7501/j.issn.0253-2670.2020.06.010 | Non-English language |
| Luo, M., et al. | https://dx.doi.org/10.1172/jci.insight.139024 | Excluded publication type or study design, or letter or comment with no relevant primary data, No comparator of interest |
| Lupia, T., et al. | https://dx.doi.org/10.1016/j.jgar.2020.02.021 | Excluded publication type or study design, or letter or comment with no relevant primary data |
| Luz, P., et al. | https://dx.doi.org/10.20344/amp.13766 | Non-English language |
| Lv, Z., et al. | https://dx.doi.org/10.1016/j.micinf.2020.05.007 | No outcome of interest |
| Ma, J., et al. | https://dx.doi.org/10.1016/j.jinf.2020.04.006 | Excluded publication type or study design, or letter or comment with no relevant primary data, No comparator of interest |
| Madapusi Balaji, T., et al. | https://dx.doi.org/10.1016/j.mehy.2020.109936 | Excluded publication type or study design, or letter or comment with no relevant primary data |
| Mahase, E. | https://dx.doi.org/10.1136/bmj.m2386 | Excluded publication type or study design, or letter or comment with no relevant primary data |
| Malard, F., et al. | http://dx.doi.org/10.1016/S2352-3026%2820%2930124-1 | Excluded publication type or study design, or letter or comment with no relevant primary data |
| Mani, V. R., et al. | https://dx.doi.org/10.2196/20548 | No exposure of interest |
| Mannheim, J., et al. | https://dx.doi.org/10.1093/jpids/piaa070 | Excluded publication type or study design, or letter or comment with no relevant primary data |
| Mareev, Y. V., et al. | https://dx.doi.org/10.18087/cardio.2020.4.n1122 | Non-English language |
| Martin-Moro, F., et al. | https://dx.doi.org/10.1111/bjh.16801 | No comparator of interest |
| McKeigue, P. M., et al. | <https://doi.org/10.1101/2020.05.28.20115394> | No exposure of interest |
| Mehta, A. K., et al. | https://dx.doi.org/10.1001/jamaoncol.2020.1938 | Excluded publication type or study design, or letter or comment with no relevant primary data |
| Mei, H., et al. | https://dx.doi.org/10.1016/S1470-2045(20)30238-2 | Excluded publication type or study design, or letter or comment with no relevant primary data |
| Meng, Y., et al. | https://dx.doi.org/10.1371/journal.ppat.1008520 | Excluded publication type or study design, or letter or comment with no relevant primary data |
| Meyerowitz, E. A., et al. | https://dx.doi.org/10.1097/QAD.0000000000002607 | No population of interest |
| Mian, M. S., et al. | https://dx.doi.org/10.7759/cureus.8136 | Excluded publication type or study design, or letter or comment with no relevant primary data |
| Minotti, C., et al. | https://dx.doi.org/10.1016/j.jinf.2020.04.026 | Excluded publication type or study design, or letter or comment with no relevant primary data |
| Mishra, V., et al. | https://dx.doi.org/10.4103/ijph.IJPH_486_20 | No exposure of interest |
| Mitra, A. R., et al. | https://dx.doi.org/10.1503/cmaj.200794 | Excluded publication type or study design, or letter or comment with no relevant primary data |
| Mo, P., et al. | https://dx.doi.org/10.1093/cid/ciaa270 | No outcome of interest |
| Moiseev, S., et al. | https://dx.doi.org/10.1016/j.jinf.2020.05.053 | No comparator of interest |
| Moreira, R. D. S. | https://dx.doi.org/10.1590/0102-311x00080020 | Excluded publication type or study design, or letter or comment with no relevant primary data |
| Moriconi, D., et al. | https://dx.doi.org/10.1016/j.orcp.2020.05.009 | Excluded publication type or study design, or letter or comment with no relevant primary data |
| Moschovas, M. C., et al. | https://dx.doi.org/10.1016/j.eururo.2020.04.028 | Excluded publication type or study design, or letter or comment with no relevant primary data |
| Moschovas, M. C., et al. | https://dx.doi.org/10.1016/j.euf.2020.05.005 | Excluded publication type or study design, or letter or comment with no relevant primary data |
| Moujaess, E., et al. | https://dx.doi.org/10.1016/j.critrevonc.2020.102972 | Excluded publication type or study design, or letter or comment with no relevant primary data |
| Mucientes Rasilla, J., et al. | https://dx.doi.org/10.1016/j.remn.2020.04.004 | Non-English language |
| Murk, J., et al. | <https://pubmed.ncbi.nlm.nih.gov/32391997/> | Non-English language |
| Myers, L. C., et al. | http://dx.doi.org/10.1001/jama.2020.7202 | No outcome of interest |
| Nayak, A., et al. | https://dx.doi.org/10.1101/2020.04.10.20060962 | Excluded publication type or study design, or letter or comment with no relevant primary data |
| Nerina, D., et al. | https://dx.doi.org/10.1016/j.oraloncology.2020.104801 | Excluded publication type or study design, or letter or comment with no relevant primary data, No population of interest |
| Ning, M. S., et al. | https://dx.doi.org/10.1016/j.radonc.2020.05.037 | No comparator of interest |
| No authors listed. | <https://doi.org/10.1158/2159-8290.cd-nb2020-032> | Excluded publication type or study design, or letter or comment with no relevant primary data |
| No authors listed. | <https://doi.org/10.1136/bmj.m1270> | Excluded publication type or study design, or letter or comment with no relevant primary data |
| No authors listed. | <https://dx.doi.org/10.1001%2Fjama.2020.7681> | Excluded publication type or study design, or letter or comment with no relevant primary data |
| No authors listed. | https://dx.doi.org/10.1111/ajt.15991 | Excluded publication type or study design, or letter or comment with no relevant primary data |
| Norredam, M., et al. | <https://pubmed.ncbi.nlm.nih.gov/32400375/> | Non-English language |
| Nunez-Torron, C., et al. | http://dx.doi.org/10.1038/s41409-020-0961-y | Excluded publication type or study design, or letter or comment with no relevant primary data |
| Ofori-Asenso, R., et al. | https://dx.doi.org/10.3332/ecancer.2020.1047 | Excluded publication type or study design, or letter or comment with no relevant primary data |
| Oh, W. K. | https://dx.doi.org/10.1016/j.annonc.2020.03.297 | Excluded publication type or study design, or letter or comment with no relevant primary data |
| Omarini, C., et al. | https://dx.doi.org/10.1016/j.ejca.2020.04.034 | Excluded publication type or study design, or letter or comment with no relevant primary data |
| Onder, G., et al. | http://dx.doi.org/10.1001/jama.2020.4683 | Excluded publication type or study design, or letter or comment with no relevant primary data |
| Ortiz-Brizuela, E., et al. | https://dx.doi.org/10.24875/RIC.20000211 | No comparator of interest, No exposure of interest |
| Oualha, M., et al. | https://dx.doi.org/10.1016/j.arcped.2020.05.010 | No population of interest, No exposure of interest |
| Ouchetto, O., et al. | <https://doi.org/10.1101/2020.07.02.20145375> | Excluded publication type or study design, or letter or comment with no relevant primary data |
| Ouyang, W., et al. | https://dx.doi.org/10.1016/j.jtho.2020.05.003 | Excluded publication type or study design, or letter or comment with no relevant primary data, No comparator of interest |
| Ozyer, Y. | http://dx.doi.org/10.1017/dmp.2020.188 | Excluded publication type or study design, or letter or comment with no relevant primary data |
| Pachiega, J., et al. | https://dx.doi.org/10.1590/S1678-9946202062045 | No exposure of interest |
| Palmieri, L., et al. | https://dx.doi.org/10.1093/gerona/glaa146 | Excluded publication type or study design, or letter or comment with no relevant primary data, No exposure of interest |
| Paneesha, S., et al. | http://dx.doi.org/10.1016/j.leukres.2020.106366 | Excluded publication type or study design, or letter or comment with no relevant primary data |
| Parohan, M., et al. | https://dx.doi.org/10.1080/13685538.2020.1774748 | Excluded publication type or study design, or letter or comment with no relevant primary data |
| Parri, N., et al. | https://dx.doi.org/10.1007/s00431-020-03683-8 | No population of interest, No exposure of interest |
| Pasquariello, P., et al. | https://dx.doi.org/10.1007/s00038-020-01399-y | Excluded publication type or study design, or letter or comment with no relevant primary data |
| Patel, N. A. | https://dx.doi.org/10.1016/j.amjoto.2020.102573 | Excluded publication type or study design, or letter or comment with no relevant primary data |
| Patel, R., et al. | <https://pubmed.ncbi.nlm.nih.gov/32405629/> | Excluded publication type or study design, or letter or comment with no relevant primary data |
| Peeters, M., et al. | https://dx.doi.org/10.1136/esmoopen-2020-000817 | Excluded publication type or study design, or letter or comment with no relevant primary data |
| Pellino, G., et al. | https://dx.doi.org/10.1097/DCR.0000000000001685 | Excluded publication type or study design, or letter or comment with no relevant primary data |
| Peng, S., et al. | https://dx.doi.org/10.1016/j.jtcvs.2020.04.005 | Excluded publication type or study design, or letter or comment with no relevant primary data |
| Pericleous, S., et al. | https://dx.doi.org/10.1097/MPA.0000000000001594 | Excluded publication type or study design, or letter or comment with no relevant primary data |
| Perini, G. F., et al. | https://dx.doi.org/10.1016/j.htct.2020.04.002 | Excluded publication type or study design, or letter or comment with no relevant primary data |
| Petrilli, C. M., et al. | <https://doi.org/10.1101/2020.04.08.20057794> | No outcome of interest, Preprint subsequently published |
| Petrilli, C. M., et al. | https://dx.doi.org/10.1136/bmj.m1966 | No outcome of interest |
| Piva, S., et al. | http://dx.doi.org/10.1016/j.jcrc.2020.04.004 | Excluded publication type or study design, or letter or comment with no relevant primary data, No exposure of interest |
| Poortmans, P. M., et al. | https://dx.doi.org/10.1016/S0140-6736(20)31240-X | Excluded publication type or study design, or letter or comment with no relevant primary data |
| Porcheddu, R., et al. | https://dx.doi.org/10.3855/jidc.12600 | Excluded publication type or study design, or letter or comment with no relevant primary data |
| Price-Haywood, E. G., et al. | https://dx.doi.org/10.1056/NEJMsa2011686 | Excluded publication type or study design, or letter or comment with no relevant primary data |
| Qin, C., et al. | https://dx.doi.org/10.1161/STROKEAHA.120.030365 | Excluded publication type or study design, or letter or comment with no relevant primary data |
| Qin, L., et al. | https://dx.doi.org/10.1002/jmv.26137 | Excluded publication type or study design, or letter or comment with no relevant primary data, No exposure of interest |
| Rassy, E., et al. | https://dx.doi.org/10.2217/fon-2020-0312 | Excluded publication type or study design, or letter or comment with no relevant primary data |
| Ravi, K. | https://dx.doi.org/10.1016/S0140-6736(20)31423-9 | Excluded publication type or study design, or letter or comment with no relevant primary data |
| Razanamahery, J., et al. | https://dx.doi.org/10.1016/j.jinf.2020.05.075 | No population of interest |
| Rees, E. M., et al. | <https://doi.org/10.1101/2020.04.30.20084780> | Excluded publication type or study design, or letter or comment with no relevant primary data |
| Rentsch, C. T., et al. | <https://doi.org/10.1101/2020.04.09.20059964> | Excluded publication type or study design, or letter or comment with no relevant primary data, No outcome of interest |
| Richardson, S., et al. | https://dx.doi.org/10.1001/jama.2020.6775 | Excluded publication type or study design, or letter or comment with no relevant primary data, No outcome of interest |
| Riva, G., et al. | https://dx.doi.org/10.1016/j.oraloncology.2020.104835 | Excluded publication type or study design, or letter or comment with no relevant primary data |
| Rivera, A., et al. | https://dx.doi.org/10.1016/j.adro.2020.03.006 | Excluded publication type or study design, or letter or comment with no relevant primary data |
| Roberto, M., et al. | https://dx.doi.org/10.1016/j.ejca.2020.05.003 | Excluded publication type or study design, or letter or comment with no relevant primary data |
| Robinson, A. G., et al. | https://dx.doi.org/10.1038/s41571-020-0394-y | Excluded publication type or study design, or letter or comment with no relevant primary data |
| Rod, J. E., et al. | https://dx.doi.org/10.11606/s1518-8787.2020054002481 | Excluded publication type or study design, or letter or comment with no relevant primary data |
| Rodler, S., et al. | https://dx.doi.org/10.1016/j.ejca.2020.04.003 | Excluded publication type or study design, or letter or comment with no relevant primary data |
| Rodriguez-Morales, A. J., et al. | https://dx.doi.org/10.1016/j.tmaid.2020.101623 | Excluded publication type or study design, or letter or comment with no relevant primary data |
| Rogado, J., et al. | https://dx.doi.org/10.1016/j.lungcan.2020.05.034 | No comparator of interest, No exposure of interest |
| Rojo, J. M. C., et al. | <https://doi.org/10.1101/2020.05.24.20111971> | Excluded publication type or study design, or letter or comment with no relevant primary data, No outcome of interest |
| Rossoff, J., et al. | https://dx.doi.org/10.1002/pbc.28504 | Excluded publication type or study design, or letter or comment with no relevant primary data, No population of interest |
| Ruggiero, A., et al. | https://dx.doi.org/10.1038/s41390-020-0919-1 | Excluded publication type or study design, or letter or comment with no relevant primary data |
| Ruiz-Patino, A., et al. | https://dx.doi.org/10.1200/GO.20.00156 | Excluded publication type or study design, or letter or comment with no relevant primary data, No outcome of interest |
| Russell, B., et al. | <https://doi.org/10.1101/2020.05.12.20094219> | Excluded publication type or study design, or letter or comment with no relevant primary data, No comparator of interest |
| Sabri, A., et al. | https://dx.doi.org/10.20452/pamw.15422 | Excluded publication type or study design, or letter or comment with no relevant primary data, No exposure of interest |
| Sahu, K. K., et al. | https://dx.doi.org/10.1200/OP.20.00167 | Excluded publication type or study design, or letter or comment with no relevant primary data |
| Sahu, K. K., et al. | https://dx.doi.org/10.1080/17474086.2020.1787147 | Excluded publication type or study design, or letter or comment with no relevant primary data |
| Saini, K. S., et al. | https://dx.doi.org/10.1016/S2352-3026(20)30123-X | Excluded publication type or study design, or letter or comment with no relevant primary data |
| Sajid, M. I., et al. | https://dx.doi.org/10.1002/pbc.28527 | Excluded publication type or study design, or letter or comment with no relevant primary data |
| Salerno, M., et al. | https://dx.doi.org/10.3390/jcm9051472 | Excluded publication type or study design, or letter or comment with no relevant primary data |
| Saluja, M., et al. | <https://pubmed.ncbi.nlm.nih.gov/32602675/> | Excluded publication type or study design, or letter or comment with no relevant primary data, No exposure of interest |
| Sanchez, L. R., et al. | https://dx.doi.org/10.1590/S1677-5538.IBJU.2020.S106 | Excluded publication type or study design, or letter or comment with no relevant primary data |
| Sanyaolu, A., et al. | http://dx.doi.org/10.1007/s42399-020-00363-4 | Excluded publication type or study design, or letter or comment with no relevant primary data |
| Sereno, M., et al. | <https://doi.org/10.1016/j.lungcan.2020.04.029> | Excluded publication type or study design, or letter or comment with no relevant primary data |
| Shah, S. J., et al. | <https://doi.org/10.1016/j.eclinm.2020.100518> | Excluded publication type or study design, or letter or comment with no relevant primary data, No outcome of interest |
| Shah, V., et al. | https://dx.doi.org/10.1111/bjh.16935 | No comparator of interest, No population of interest |
| Shao, F., et al. | https://dx.doi.org/10.1016/j.resuscitation.2020.04.005 | Excluded publication type or study design, or letter or comment with no relevant primary data, No exposure of interest |
| Sharma, A., et al. | https://dx.doi.org/10.1111/dth.13778 | Excluded publication type or study design, or letter or comment with no relevant primary data |
| Sharpless, N. E. | https://dx.doi.org/10.1126/science.abd3377 | Excluded publication type or study design, or letter or comment with no relevant primary data |
| Shekerdemian, L. S., et al. | https://dx.doi.org/10.1001/jamapediatrics.2020.1948 | No population of interest |
| Shen, Y., et al. | https://dx.doi.org/10.1080/22221751.2020.1787103 | Excluded publication type or study design, or letter or comment with no relevant primary data, No exposure of interest |
| Shi, Q., et al. | https://dx.doi.org/10.2337/dc20-0598 | No population of interest |
| Shi, Y., et al. | http://dx.doi.org/10.1186/s13054-020-2833-7 | No outcome of interest, Excluded publication type or study design, or letter or comment with no relevant primary data |
| Sidaway, P. | https://dx.doi.org/10.1038/s41571-020-0366-2 | Excluded publication type or study design, or letter or comment with no relevant primary data |
| Silva, C. A., et al. | https://dx.doi.org/10.6061/clinics/2020/e1931 | Excluded publication type or study design, or letter or comment with no relevant primary data |
| Singh, A. K., et al. | https://dx.doi.org/10.1016/j.dsx.2020.03.016 | Excluded publication type or study design, or letter or comment with no relevant primary data |
| Singh, A. K., et al. | https://dx.doi.org/10.1111/dom.14124 | Excluded publication type or study design, or letter or comment with no relevant primary data |
| Singhal, T. | https://dx.doi.org/10.1111/dom.14124 | Excluded publication type or study design, or letter or comment with no relevant primary data |
| Sinha, I. P., et al. | https://dx.doi.org/10.1016/S2213-2600(20)30152-1 | Excluded publication type or study design, or letter or comment with no relevant primary data |
| Siordia, J. A., Jr. | https://dx.doi.org/10.1016/j.jcv.2020.104357 | Excluded publication type or study design, or letter or comment with no relevant primary data |
| Sisó-Almirall, A., et al. | <https://doi.org/10.1101/2020.06.18.20134510> | No outcome of interest |
| Slone, J. S., et al. | https://dx.doi.org/10.1080/08880018.2020.1772913 | Excluded publication type or study design, or letter or comment with no relevant primary data |
| Smith-Ray, R., et al. | https://dx.doi.org/10.2196/19606 | No population of interest, Excluded publication type or study design, or letter or comment with no relevant primary data |
| Solodky, M. L., et al. | <https://doi.org/10.1016/j.annonc.2020.04.475> | No outcome of interest, Excluded publication type or study design, or letter or comment with no relevant primary data |
| Soran, A., et al. | https://dx.doi.org/10.5152/ejbh.2020.240320 | Excluded publication type or study design, or letter or comment with no relevant primary data |
| Sousa, G. J. B., et al. | https://dx.doi.org/10.1017/S0950268820001405 | No exposure of interest |
| Ssentongo, P., et al. | <https://doi.org/10.1101/2020.05.10.20097253> | Excluded publication type or study design, or letter or comment with no relevant primary data |
| Strain, W. D., et al. | <https://doi.org/10.1101/2020.05.05.20091967> | No population of interest, Excluded publication type or study design, or letter or comment with no relevant primary data |
| Su, Q., et al. | <https://doi.org/10.1101/2020.06.23.20136200> | Excluded publication type or study design, or letter or comment with no relevant primary data |
| Suarez, V., et al. | https://dx.doi.org/10.1016/j.rce.2020.05.007 | No exposure of interest, Non-English language |
| Suleyman, G., et al. | https://dx.doi.org/10.1001/jamanetworkopen.2020.12270 | No outcome of interest |
| Sun, L., et al. | https://dx.doi.org/10.1002/jmv.25966 | Excluded publication type or study design, or letter or comment with no relevant primary data, No exposure of interest |
| Tambe, M. P., et al. | https://dx.doi.org/10.4103/ijph.IJPH_522_20 | Excluded publication type or study design, or letter or comment with no relevant primary data, No exposure of interest |
| Tang, L. V., et al. | https://dx.doi.org/10.1016/S1470-2045(20)30311-9 | Excluded publication type or study design, or letter or comment with no relevant primary data |
| Taylor, E. H., et al. | http://dx.doi.org/10.36303/SAJAA.2020.26.3.2428 | Excluded publication type or study design, or letter or comment with no relevant primary data |
| Thai, P. Q., et al. | https://dx.doi.org/10.1017/S0950268820001259 | No exposure of interest |
| Tian, S., et al. | http://dx.doi.org/10.1093/ofid/ofaa152 | No population of interest, Excluded publication type or study design, or letter or comment with no relevant primary data |
| Tian, W., et al. | https://dx.doi.org/10.1002/jmv.26050 | Excluded publication type or study design, or letter or comment with no relevant primary data |
| Trapani, D., et al. | https://dx.doi.org/10.1016/j.ejca.2020.04.017 | No comparator of interest, Excluded publication type or study design, or letter or comment with no relevant primary data |
| Udwadia, Z. F., et al. | <https://pubmed.ncbi.nlm.nih.gov/32602683/> | Excluded publication type or study design, or letter or comment with no relevant primary data |
| Ujjan, I. D., et al. | https://dx.doi.org/10.12669/pjms.36.COVID19-S4.2829 | No exposure of interest |
| Urra, J. M., et al. | https://dx.doi.org/10.1016/j.clim.2020.108486 | No outcome of interest |
| Valenza, F., et al. | https://dx.doi.org/10.1177/0300891620923790 | Excluded publication type or study design, or letter or comment with no relevant primary data, No comparator of interest |
| Van der Moeren, N., et al. | <https://pubmed.ncbi.nlm.nih.gov/32391998/> | Non-English language |
| Vasquez, L., et al. | https://dx.doi.org/10.1016/S1470-2045(20)30280-1 | Excluded publication type or study design, or letter or comment with no relevant primary data |
| Venkatesulu, B. P., et al. | <https://doi.org/10.1101/2020.05.27.20115303> | Excluded publication type or study design, or letter or comment with no relevant primary data |
| Vici, P., et al. | https://dx.doi.org/10.2217/imt-2020-0142 | Excluded publication type or study design, or letter or comment with no relevant primary data |
| Vila-Corcoles, A., et al. | <https://pubmed.ncbi.nlm.nih.gov/32588837/> | Non-English language |
| Vuagnat, P., et al. | https://dx.doi.org/10.1186/s13058-020-01293-8 | Excluded publication type or study design, or letter or comment with no relevant primary data, No comparator of interest |
| Wainer, P., et al. | <https://pubmed.ncbi.nlm.nih.gov/32442932/> | Non-English language |
| Wan, S., et al. | https://dx.doi.org/10.1002/jmv.25783 | Excluded publication type or study design, or letter or comment with no relevant primary data |
| Wang, B., et al. | https://dx.doi.org/10.18632/aging.103000 | Excluded publication type or study design, or letter or comment with no relevant primary data |
| Wang, B., et al. | https://dx.doi.org/10.1016/j.critrevonc.2020.103032 | Excluded publication type or study design, or letter or comment with no relevant primary data |
| Wang, C. Z., et al. | https://dx.doi.org/10.1002/jmv.26071 | Excluded publication type or study design, or letter or comment with no relevant primary data, No exposure of interest |
| Wang, D., et al. | https://dx.doi.org/10.1001/jama.2020.1585 | Excluded publication type or study design, or letter or comment with no relevant primary data, No outcome of interest |
| Wang, H., et al. | https://dx.doi.org/10.1016/S1470-2045(20)30149-2 | Excluded publication type or study design, or letter or comment with no relevant primary data |
| Wang, L., et al. | <https://doi.org/10.1159/000507471> | No outcome of interest |
| Wang, L., et al. | https://dx.doi.org/10.1183/13993003.00398-2020 | Excluded publication type or study design, or letter or comment with no relevant primary data |
| Wang, X., et al. | https://dx.doi.org/10.34133/2020/2402961 | Excluded publication type or study design, or letter or comment with no relevant primary data |
| Wang, Y., et al. | http://dx.doi.org/10.1093/infdis/jiaa119 | Excluded publication type or study design, or letter or comment with no relevant primary data, No exposure of interest |
| Wang, Z., et al. | https://dx.doi.org/10.1093/cid/ciaa272 | Excluded publication type or study design, or letter or comment with no relevant primary data, No outcome of interest |
| Wiersinga, W. J. | <https://pubmed.ncbi.nlm.nih.gov/32406637/> | Non-English language |
| Williamson, E. et al. | <https://doi.org/10.1101/2020.05.06.20092999> | No population of interest, Excluded publication type or study design, or letter or comment with no relevant primary data |
| Wise, J. | https://dx.doi.org/10.1136/bmj.m1735 | Excluded publication type or study design, or letter or comment with no relevant primary data |
| Woo, S. H., et al. | <https://doi.org/10.1101/2020.07.16.20155739> | Excluded publication type or study design, or letter or comment with no relevant primary data, No outcome of interest |
| Wu, J., et al. | https://dx.doi.org/10.1111/joim.13063 | Excluded publication type or study design, or letter or comment with no relevant primary data, No outcome of interest |
| Wu, Z., et al. | http://dx.doi.org/10.1001/jama.2020.2648 | Excluded publication type or study design, or letter or comment with no relevant primary data |
| Wynants L., et al. | <https://doi.org/10.1136/bmj.m1328> | Excluded publication type or study design, or letter or comment with no relevant primary data |
| Wyper, G. M. A., et al. | https://dx.doi.org/10.1186/s13690-020-00433-y | No population of interest |
| Xia, Y., et al. | https://dx.doi.org/10.1016/S1470-2045(20)30150-9 | Excluded publication type or study design, or letter or comment with no relevant primary data |
| Xu, G., et al. | https://dx.doi.org/10.1007/s12016-020-08792-8 | Excluded publication type or study design, or letter or comment with no relevant primary data |
| Xu, L., et al. | <https://doi.org/10.1101/2020.03.30.20047415> | Excluded publication type or study design, or letter or comment with no relevant primary data |
| Yadav, S. P. | https://dx.doi.org/10.1097/MPH.0000000000001872 | Excluded publication type or study design, or letter or comment with no relevant primary data, No population of interest |
| Yan C.H., et al. | <https://doi.org/10.1002/alr.22579> | No outcome of interest, No population of interest |
| Yan, F., et al. | https://dx.doi.org/10.1002/hed.26209 | Excluded publication type or study design, or letter or comment with no relevant primary data |
| Yan, S., et al. | https://dx.doi.org/10.1097/CEJ.0000000000000605 | Excluded publication type or study design, or letter or comment with no relevant primary data |
| Yang L., et al. | <https://doi.org/10.1016/j.jcv.2020.104475> | Excluded publication type or study design, or letter or comment with no relevant primary data |
| Yang, J., et al. | https://dx.doi.org/10.1016/j.ijid.2020.03.017 | Excluded publication type or study design, or letter or comment with no relevant primary data |
| Yang, K., et al. | https://dx.doi.org/10.1016/S1470-2045(20)30310-7 | Excluded publication type or study design, or letter or comment with no relevant primary data, No comparator of interest |
| Yanover, C., et al. | <https://doi.org/10.1101/2020.05.07.20091652> | No outcome of interest |
| Yu, J., et al. | <https://doi.org/10.1001/jamaoncol.2020.0980> | Excluded publication type or study design, or letter or comment with no relevant primary data |
| Yu, Y., et al. | http://dx.doi.org/10.1186/s13054-020-02939-x | Excluded publication type or study design, or letter or comment with no relevant primary data |
| Zachariah, P., et al. | https://dx.doi.org/10.1001/jamapediatrics.2020.2430 | No population of interest |
| Zadori, N., et al. | https://dx.doi.org/10.1007/s00134-020-06161-9 | Excluded publication type or study design, or letter or comment with no relevant primary data |
| Zaki, N., et al. | <https://doi.org/10.1101/2020.06.16.20132639> | Excluded publication type or study design, or letter or comment with no relevant primary data |
| Zaki, N., et al. | <https://doi.org/10.1101/2020.06.18.20134478> | Excluded publication type or study design, or letter or comment with no relevant primary data |
| Zeng, L., et al. | <https://doi.org/10.1101/2020.03.25.20043166> | No outcome of interest |
| Zhang, G., et al. | https://dx.doi.org/10.1016/j.jcv.2020.104364 | No outcome of interest |
| Zhang, J. J., et al. | https://dx.doi.org/10.1111/all.14238 | Excluded publication type or study design, or letter or comment with no relevant primary data, No exposure of interest |
| Zhang, J., et al. | https://dx.doi.org/10.3389/fpubh.2020.00264 | Excluded publication type or study design, or letter or comment with no relevant primary data, No outcome of interest |
| Zhang, N., et al. | <https://doi.org/10.1007/s00330-020-06955-x> | Excluded publication type or study design, or letter or comment with no relevant primary data, No exposure of interest |
| Zhao, N., et al. | <https://doi.org/10.3779/j.issn.1009-3419.2020.102.15> | Non-English language |
| Zheng, Y., et al. | https://dx.doi.org/10.1016/j.phrs.2020.104821 | No population of interest, No outcome of interest |
| Zheng, Z., et al. | https://dx.doi.org/10.1016/j.jinf.2020.04.021 | Excluded publication type or study design, or letter or comment with no relevant primary data |
| Ziehr, D. R., et al. | https://dx.doi.org/10.1164/rccm.202004-1163LE | Excluded publication type or study design, or letter or comment with no relevant primary data |

Supplementary Table 5**: Characteristics of included studies omitted from meta-analyses due to overlapping, insufficient or inconsistent data**

| **Study** | **Publication type** | **Population** | | | | | **Exposure (Cancer)** | | | **Comparator** | | **Minimum Follow up (days)** | **Reason for omission from analysis** |
| --- | --- | --- | --- | --- | --- | --- | --- | --- | --- | --- | --- | --- | --- |
|  |  | **Description** | **N** | **Age (median years)** | **Male (%)** | **Method of COVID-19-19 diagnosis** | **Cancer status** | **Cancer type** | **N** | **Definition** | **N** |  |  |
| **China** | | | | | | | | | | | | |  |
| Asfahan S.[[11](#_ENREF_11)] | Original journal article | All COVID-19 patients | 20812 | NR | NR | PCR assay | NR | NR | 107 | No cancer | 20705 | 0 | Overlapping data |
| Bai T.[[12](#_ENREF_12)] | Preprint | Hospital inpatients | 127 | 55 | 63.0 | PCR assay | NR | NR | 5 | No cancer | 122 | 10 | Overlapping data |
| Cao J.[[13](#_ENREF_13)] | Original journal article | Hospital inpatients | 102 | 54 | 52 | PCR assay | NR | NR | 4 | No cancer | 98 | 14 | Overlapping data |
| Chen T, Dai Z.[[14](#_ENREF_14)] | Original journal article | Hospital inpatients | 55 | 74 [100% ≥ 65] | 61.8 | PCR assay | NR | NR | 5 | No cancer | 50 | 10 | Overlapping data |
| Chen T, Wu D.[[15](#_ENREF_15)] | Original journal article | Hospital inpatients | 274 | 62 | 62.4 | PCR assay | NR | NR | 7 | No cancer | 267 | 16 | Overlapping data |
| Chen X.[[16](#_ENREF_16)] | Preprint | Hospital inpatients | 291 | 46 | 49.8 | PCR assay | NR | NR | 2 | No cancer | 289 | 5 | Overlapping data |
| Chen N.[[17](#_ENREF_17)] | Original journal article | Hospital inpatients | 99 | 55.5* | 67.7 | PCR assay | NR | NR | 1 | No cancer | 98 | 6 | Overlapping data |
| Dai M.[[18](#_ENREF_18)] | Original journal article | Hospital inpatients | 641 | 64 | 47.1 | Unclear | Any | Mixed | 105 | No cancer | 536 | 0 | Insufficient or inconsistent data |
|  |  |  | 545 | NR | NR | Unclear | Any | Haematological | 9 | No cancer | 536 | 0 |  |
|  |  |  | 571 | NR | NR | Unclear | Active | Mixed | 35 | No cancer | 536 | 0 |  |
| Deng G.[[19](#_ENREF_19)] | Letter or commentary | All COVID-19 patients | 20982 | NR | NR | PCR assay | NR | NR | 107 | No cancer | 20875 | 0 | Overlapping data |
| Deng Y.[[20](#_ENREF_20)] | Original journal article | Hospital inpatients | 225 | NR | 55.1 | PCR assay | NR | NR | 8 | No cancer | 217 | 0 | Overlapping data |
| Ding M.[[21](#_ENREF_21)] | Original journal article | Hospital inpatients | 26 | 68 | 50 | Unclear | NR | Bowel | 1 | No cancer | 25 | 0 | Overlapping data |
| Du R.H.[[22](#_ENREF_22)] | Original journal article | Hospital inpatients | 179 | 57.6* | 54.2 | Unclear | NR | NR | 4 | No cancer | 175 | 0 | Overlapping data |
| Gan J.[[23](#_ENREF_23)] | Original journal article | Hospital inpatients | 95 | 65 | 61 | PCR assay or serology | NR | NR | 6 | No cancer | 89 | 1 | Overlapping data |
| Hu B.[[24](#_ENREF_24)] | Preprint | Hospital inpatients with critical disease | 50 | 62 | 68 | PCR assay | NR | NR | 2 | No cancer | 48 | 3 | Overlapping data |
| Hu H.[[25](#_ENREF_25)] | Original journal article | Hospital inpatients with critical disease | 105 | 60.8* | 50.9 | Unclear | NR | NR | 6 | No cancer | 99 | 0 | Overlapping data |
| Huang R.[[26](#_ENREF_26)] | Original journal article | Hospital inpatients | 202 | 44 | 57.4 | PCR assay | NR | NR | 2 | No cancer | 200 | 0 | Overlapping data |
| Huang J.[[27](#_ENREF_27)] | Original journal article | Hospital inpatients | 299 | 53.4* | 53.5 | PCR assay | NR | NR | 9 | No cancer | 290 | 0 | Overlapping data |
| Lei S.[[28](#_ENREF_28)] | Original journal article | Surgical patients | 34 | 55 | 41.2 | PCR assay | NR | NR | 9 | No cancer | 25 | 33 | Overlapping data |
| Li L.[[29](#_ENREF_29)] | Letter or commentary | Hospital inpatients with critical disease | 82 | NR | NR | NR | NR | NR | NR | No cancer | NR | 0 | Insufficient or inconsistent data |
| Lian J.[[30](#_ENREF_30)] | Original journal article | Hospital inpatients | 465 | 45 | 52.3 | PCR assay | NR | NR | 5 | No cancer | 460 | 0 | Overlapping data |
| Ma S.[[31](#_ENREF_31)] | Original journal article | Hospital inpatients with critical disease | 93 | 67 | 54.8 | PCR assay or clinical/imaging | NR | NR | 2 | No cancer | 91 | NR | Overlapping data |
| Ruan Q.[[32](#_ENREF_32)] | Letter or commentary | Hospital inpatients | 150 | NR | 68.0 | PCR assay | NR | NR | 3 | No cancer | 147 | NR | Overlapping data |
| Shang Y.[[33](#_ENREF_33)] | Original journal article | Hospital inpatients with severe disease | 113 | 66 | 64.6 | PCR assay | NR | NR | 8 | No cancer | 105 | 3 | Overlapping data |
| Shi H.[[34](#_ENREF_34)] | Original journal article | Hospital inpatients | 81 | 49.5* | 51.9 | PCR assay | NR | NR | 4 | No cancer | 77 | 15 | Overlapping data |
| Shi S.[[35](#_ENREF_35)] | Original journal article | Hospital inpatients | 416 | 64 | 49.3 | PCR assay | NR | NR | 9 | No cancer | 407 | 4 | Overlapping data |
| Wang K.[[36](#_ENREF_36)] | Letter or commentary | Hospital inpatients | 548 | NR | 50.9 | NR | NR | NR | 24 | No cancer | 524 | 15 | Overlapping data |
| World Health Organization[[37](#_ENREF_37)] | Report | All COVID-19 patients | 55924 | 51 | 51.1 | PCR assay | NR | NR | NR | All COVID-19 cases in a population | 55924 | 0 | Insufficient or inconsistent data |
| Xu P.P.[[38](#_ENREF_38)] | Original journal article | Hospital inpatients | 703 | 46.1* | 54.3 | PCR assay | NR | NR | 9 | No cancer | 694 | 0 | Overlapping data |
| Yang X.[[39](#_ENREF_39)] | Original journal article | Hospital inpatients with critical disease | 52 | 59.7* | 67.3 | PCR assay | NR | NR | 2 | No cancer | 50 | 13 | Overlapping data |
| Ye C.[[40](#_ENREF_40)] | Original journal article | Hospital inpatients | 856 | 46 | 51.3 | Unclear | NR | NR | 8 | No cancer | 848 | 0 | Overlapping data |
| Yin Y.[[41](#_ENREF_41)] | Preprint | Hospital inpatients with critical disease | 112 | 66 | 68.7 | PCR assay or clinical/imaging | NR | NR | 4 | No cancer | 108 | 28 | Overlapping data |
| Yuan M.[[42](#_ENREF_42)] | Original journal article | Hospital inpatients | 27 | 60 | 44.4 | PCR assay | NR | NR | 1 | No cancer | 26 | 0 | Overlapping data |
| Zeng H.[[43](#_ENREF_43)] | Preprint | Hospital inpatients | 1031 | 60 | 52.2 | Unclear | NR | NR | 29 | No cancer | 1002 | 21 | Overlapping data |
| Zhang J.[[44](#_ENREF_44)] | Original journal article | Hospital inpatients | 111 | 38 | 41.1 | PCR assay | NR | NR | 8 | No cancer | 103 | 9 | Overlapping data |
| Zhang S, Guo M.[[45](#_ENREF_45)] | Preprint | Hospital inpatients | 315 | 57 | 55.6 | PCR assay or clinical/imaging | NR | NR | 12 | No cancer | 303 | 28 | Overlapping data |
| Zhang S, Zhao J.[[46](#_ENREF_46)] | Preprint | Hospital inpatients | 262 | 55 | 60.3 | Unclear | NR | NR | 10 | No cancer | 252 | 0 | Overlapping data |
| Zhou F.[[47](#_ENREF_47)] | Original journal article | Hospital inpatients | 191 | 56 | 62.3 | PCR assay | NR | NR | 2 | No cancer | 189 | NR | Overlapping data |
| Zou X.[[48](#_ENREF_48)] | Original journal article | Hospital inpatients with critical disease | 154 | 60.7* | 43.5 | PCR assay | NR | NR | 5 | No cancer | 149 | 14 | Overlapping data |
| **Iran** | | | | | | | | | | | | |  |
| Norooznezhad A.[[49](#_ENREF_49)] | Original journal article | Hospital inpatients | 416 | NR [19.7% ≥ 65] | NR | PCR assay | NR | NR | NR | No cancer | NR | 0 | Insufficient or inconsistent data |
| **Italy** | | | | | | | | | | | | |  |
| Gallo O.[[50](#_ENREF_50)] | Letter or commentary | Hospital inpatients | 119 | 73 (cancer) 64 (non- cancer) | 61.3 | NR | NR | Mixed | 18 | No cancer | 101 | 0 | Insufficient or inconsistent data |
| **UK** | | | | | | | | | | | | |  |
| Tomlins J.[[51](#_ENREF_51)] | Letter or commentary | Hospital inpatients | 95 | 75 | 63.2 | Unclear | NR | NR | 20 | No cancer | 75 | 6 | Overlapping data |

NR = not reported; N = number of individuals; PCR = Polymerase chain reaction

*Mean

Supplementary Table 6**:** **Factors included in adjustment of ORs and HRs for comparisons between those with and without pre-existing cancer**

| Study | Age | Sex | Race | Region | Smoking | Alcohol | Number of comorbidities | HT | DM | CHD | CVD | Obesity/  BMI | Liver disease | Asthma/ lung disease/ COPD | Kidney disease | Immune-deficiencies | Neurological disease/ Dementia | Haematologi -cal disease | Anti-viral treatment | Pneumonia | Vital signs | Laboratory and/or clinical results |
| --- | --- | --- | --- | --- | --- | --- | --- | --- | --- | --- | --- | --- | --- | --- | --- | --- | --- | --- | --- | --- | --- | --- |
| Cancer vs no cancer adj OR studies | | | | | | | | | | | | | | | | | | | | | | |
| Mehta*[[52](#_ENREF_52)] | X | X |  |  |  |  |  |  |  |  |  |  |  |  |  |  |  |  |  |  |  |  |
| Reilev[[53](#_ENREF_53)] | X | X |  |  |  |  |  |  |  |  |  |  |  |  |  |  |  |  |  |  |  |  |
| Mendy[[54](#_ENREF_54)] | X | X | X |  | X |  |  |  |  |  |  |  |  |  |  |  |  |  |  |  |  |  |
| Iftimie[[55](#_ENREF_55)] | X | X |  |  | X | X |  |  |  |  |  |  |  |  |  |  |  |  |  |  |  |  |
| Stroppa*[[56](#_ENREF_56)] | X | X |  |  |  |  |  |  |  |  |  |  |  |  |  |  |  |  | X | X |  |  |
| Tian*[[2](#_ENREF_2)] | X | X |  |  |  |  |  | X | X | X | X |  | X | X COPD | X |  |  |  |  |  |  |  |
| Heili-Frades[[57](#_ENREF_57)] | X |  |  |  | X |  |  | X | X | X |  |  |  | X | X |  | X |  |  |  |  |  |
| Pérez-Tanoira[[58](#_ENREF_58)] | X |  |  |  |  |  |  | X | X | X |  |  |  | X COPD | X | X |  |  |  |  |  | Lymphocytes, LDH |
| Peixoto[[59](#_ENREF_59)] | X | X |  | X |  |  |  |  | X | X |  |  | X | X | X | X | X | X |  |  |  |  |
| Cancer vs no cancer adj HR studies | | | | | | | | | | | | | | | | | | | | | | |
| Xie[[3](#_ENREF_3)] | X | X |  |  | X |  |  | X |  |  |  |  |  |  |  |  |  |  |  |  |  | APACHE II score, SOFA score and possibly P/F ratio, AST, LDH, CRP, D-dimer, TnI, albumin, creatinine, INR, lymphocytes |
| Zhao[[60](#_ENREF_60)] | X |  |  |  |  |  | ? | X | X | X | X |  | X | X | X |  |  |  |  |  |  |  |
| Rossi[[61](#_ENREF_61)] | X | X |  |  |  |  |  |  |  |  |  |  |  |  |  |  |  |  |  |  |  |  |
| Lee[[62](#_ENREF_62)] | X | X |  |  |  |  |  | X | X | X | X |  |  | X COPD | X |  |  |  |  |  |  |  |
| Docherty[[5](#_ENREF_5)] | X | X |  |  |  |  |  |  | X | X |  | X | X | X | X |  | X |  |  |  |  |  |
| Active vs no cancer adj HR studies | | | | | | | | | | | | | | | | | | | | | | |
| Ciceri[[63](#_ENREF_63)] | X | X |  |  |  |  |  | X | X | X |  |  |  |  | X |  |  |  |  |  |  | RALE score, Lymphocyte, CRP |
| Galloway[[6](#_ENREF_6)] | X | X |  |  |  |  |  |  |  |  |  |  |  |  |  |  |  |  |  |  |  |  |
| Wang Z[[64](#_ENREF_64)] | X | X | X |  | X |  |  | X | X |  |  | X |  | X |  | X |  |  |  |  | X | Oxygen saturation, WBC, ALT, creatinine |
| Active vs no active cancer adj OR studies | | | | | | | | | | | | | | | | | | | | | | |
| COVIDSurg Collaborative[[4](#_ENREF_4)] | X | X |  |  |  |  | X |  |  |  |  |  |  |  |  |  |  |  |  |  |  | ASA grade, WBC, surgical urgency, grade of surgery, COVID-19 diagnosis, pre or post surgery |

Adj = adjusted; ALT = alanine aminotransferase; APACHE = Acute Physiology and Chronic Health Evaluation; ALT = alanine aminotransferase; ASA = American Society of Anesthesiologists; AST = aspartate aminotransferase; CHD = coronary heart disease; COPD = chronic obstructive pulmonary disease; CRP = C-reactive protein; CVD = cerebrovascular disease; DM = diabetes mellitus; HT = hypertension, INR = international normalised ratio; LDH = lactate dehydrogenase; P/F ratio = arterial oxygen partial pressure to fractional inspired oxygen ratio; RALE = Radiographic Assessment of Lung Edema; SOFA = Sequential Organ Failure Assessment; TnI = troponin I: WBC = white blood cell count

* Covariates were controlled for by matching methods

**References**

1. Higgins JP, Thompson SG. Quantifying heterogeneity in a meta-analysis. Statistics in medicine. 2002;21(11):1539-58.

2. Tian J, Yuan X, Xiao J, Zhong Q, Yang C, Liu B, et al. Clinical characteristics and risk factors associated with COVID-19 disease severity in patients with cancer in Wuhan, China: a multicentre, retrospective, cohort study. Lancet Oncol. 2020;29:29.

3. Xie J, Wu W, Li S, Hu Y, Hu M, Li J, et al. Clinical Characteristics and Outcomes of Critically Ill Patients with Novel Coronavirus Infectious Disease (COVID-19) In China: A Retrospective Multicenter Descriptive Study. [4/25/2020]. SSRN. 2020: <http://dx.doi.org/10.2139/ssrn.3588590>.

4. COVIDSurg Collaborative. Mortality and pulmonary complications in patients undergoing surgery with perioperative SARS-CoV-2 infection: an international cohort study. Lancet. 2020;29:29.

5. Docherty AB, Harrison EM, Green CA, Hardwick HE, Pius R, Norman L, et al. Features of 20 133 UK patients in hospital with covid-19 using the ISARIC WHO Clinical Characterisation Protocol: prospective observational cohort study. Bmj. 2020;369:m1985.

6. Galloway JB, Norton S, Barker RD, Brookes A, Carey I, Clarke BD, et al. A clinical risk score to identify patients with COVID-19 at high risk of critical care admission or death: An observational cohort study. Journal of Infection. 2020;29:29.

7. Kirk M, Smurthwaite K, Bräunig J, Trevenar S, D’Este C, Lucas R, et al. The PFAS Health Study: Systematic Literature Review. Canberra: The Australian National University; 2018.

8. CLARITY Group at McMaster University. Tool to Assess Risk of Bias in Cohort Studies. 2021 [Available from: <https://www.evidencepartners.com/resources/methodological-resources/tool-to-assess-risk-of-bias-in-cohort-studies-distillersr>].

9. Sterne JAC, Hernán MA, Reeves BC, Savović J, Berkman ND, Viswanathan M, et al. ROBINS-I: a tool for assessing risk of bias in non-randomised studies of interventions. Bmj. 2016;355:i4919.

10. Prostate Cancer Foundation of Australia and Cancer Council Australia PSA Testing Guidelines Expert Advisory Panel. Clinical practice guidelines PSA Testing and Early Management of Test-Detected Prostate Cancer. Sydney: Cancer Council Australia; 2016 [cited 2021 Jun 16]. Available from: <http://wiki.cancer.org.au/australiawiki/index.php?oldid=211982>].

11. Asfahan S, Deokar K, Dutt N, Niwas R, Jain P, Agarwal M. Extrapolation of mortality in COVID-19: Exploring the role of age, sex, co-morbidities and health-care related occupation. Monaldi Arch Chest Dis. 2020;90(2):21.

12. Bai T, Tu S, Wei Y, Xiao L, Jin Y, Zhang L, et al. Clinical and Laboratory Factors Predicting the Prognosis of Patients with COVID-19: An Analysis of 127 Patients in Wuhan, China. [2/26/2020]. SSRN. 2020: <http://dx.doi.org/10.2139/ssrn.3546118>.

13. Cao J, Tu WJ, Cheng W, Yu L, Liu YK, Hu X, et al. Clinical Features and Short-term Outcomes of 102 Patients with Corona Virus Disease 2019 in Wuhan, China. Clin Infect Dis. 2020;02:02.

14. Chen T, Dai Z, Mo P, Li X, Ma Z, Song S, et al. Clinical Characteristics and Outcomes of Older Patients with Coronavirus Disease 2019 (COVID-19) in Wuhan, China: A Single-Centered, Retrospective Study. The journals of gerontology Series A, Biological sciences and medical sciences. 2020;75(9):1788-95.

15. Chen T, Wu D, Chen H, Yan W, Yang D, Chen G, et al. Clinical characteristics of 113 deceased patients with coronavirus disease 2019: retrospective study. Bmj. 2020;368:m1091.

16. Chen X, Zheng F, Qing Y, Ding S, Yang D, Lei C, et al. Epidemiological and clinical features of 291 cases with coronavirus disease 2019 in areas adjacent to Hubei, China: a double-center observational study. medRxiv. 2020:2020.03.03.20030353.

17. Chen N, Zhou M, Dong X, Qu J, Gong F, Han Y, et al. Epidemiological and clinical characteristics of 99 cases of 2019 novel coronavirus pneumonia in Wuhan, China: a descriptive study. Lancet. 2020;395(10223):507-13.

18. Dai M, Liu D, Liu M, Zhou F, Li G, Chen Z, et al. Patients with Cancer Appear More Vulnerable to SARS-CoV-2: A Multicenter Study during the COVID-19 Outbreak. Cancer Discov. 2020;10(6):783-91.

19. Deng G, Yin M, Chen X, Zeng F. Clinical determinants for fatality of 44,672 patients with COVID-19. Critical Care. 2020;24 (1) (no pagination)(179).

20. Deng Y, Liu W, Liu K, Fang YY, Shang J, Zhou L, et al. Clinical characteristics of fatal and recovered cases of coronavirus disease 2019 in Wuhan, China: a retrospective study. Chin Med J. 2020;133(11):1261-7.

21. Ding M, Zhang Q, Li Q, Wu T, Huang YZ. Correlation analysis of the severity and clinical prognosis of 32 cases of patients with COVID-19. Respiratory medicine. 2020;167:105981.

22. Du RH, Liang LR, Yang CQ, Wang W, Cao TZ, Li M, et al. Predictors of mortality for patients with COVID-19 pneumonia caused by SARS-CoV-2: a prospective cohort study. Eur Respir J. 2020;55(5):05.

23. Gan J, Li J, Li S, Yang C. Leucocyte Subsets Effectively Predict the Clinical Outcome of Patients With COVID-19 Pneumonia: A Retrospective Case-Control Study. Frontiers in public health. 2020;8:299.

24. Hu B, Wang D, Hu C, Hu M, Zhu F, Xiang H, et al. Clinical features of critically ill patients with COVID-19 infection in China. [07/03/2020]. Research Square. 2020: <https://doi.org/10.21203/rs.3.rs-16250/v1>.

25. Hu H, Yao N, Qiu Y. Comparing Rapid Scoring Systems in Mortality Prediction of Critically Ill Patients With Novel Coronavirus Disease. Academic emergency medicine : official journal of the Society for Academic Emergency Medicine. 2020;27(6):461-8.

26. Huang R, Zhu L, Xue L, Liu L, Yan X, Wang J, et al. Clinical findings of patients with coronavirus disease 2019 in Jiangsu province, China: A retrospective, multi-center study. PLoS neglected tropical diseases. 2020;14(5):e0008280.

27. Huang J, Cheng A, Kumar R, Fang Y, Chen G, Zhu Y, et al. Hypoalbuminemia predicts the outcome of COVID-19 independent of age and co-morbidity. J Med Virol. 2020;14:14.

28. Lei S, Jiang F, Su W, Chen C, Chen J, Mei W, et al. Clinical characteristics and outcomes of patients undergoing surgeries during the incubation period of COVID-19 infection. EClinicalMedicine. 2020:100331.

29. Li L, Zhang B, He B, Gong Z, Chen X. Critical patients with coronavirus disease 2019: Risk factors and outcome nomogram. Journal of Infection. 2020;80(6):e37-e8.

30. Lian J, Jin X, Hao S, Jia H, Cai H, Zhang X, et al. Epidemiological, clinical, and virological characteristics of 465 hospitalized cases of coronavirus disease 2019 (COVID-19) from Zhejiang province in China. Influenza other respi. 2020;12:12.

31. Ma S, Lai X, Chen Z, Tu S, Qin K. Clinical Characteristics of Critically Ill Patients Co-infected with SARS-CoV-2 and the Influenza Virus in Wuhan, China. Int J Infect Dis. 2020;26:26.

32. Ruan Q, Yang K, Wang W, Jiang L, Song J. Clinical predictors of mortality due to COVID-19 based on an analysis of data of 150 patients from Wuhan, China. Intensive care medicine. 2020;46(5):846-8.

33. Shang Y, Liu T, Wei Y, Li J, Shao L, Liu M, et al. Scoring systems for predicting mortality for severe patients with COVID-19. EClinicalMedicine. 2020;24.

34. Shi H, Han X, Jiang N, Cao Y, Alwalid O, Gu J, et al. Radiological findings from 81 patients with COVID-19 pneumonia in Wuhan, China: a descriptive study. The Lancet Infectious diseases. 2020;20(4):425-34.

35. Shi S, Qin M, Shen B, Cai Y, Liu T, Yang F, et al. Association of Cardiac Injury With Mortality in Hospitalized Patients With COVID-19 in Wuhan, China. JAMA Cardiol. 2020;25:25.

36. Wang K, Zhang Z, Yu M, Tao Y, Xie M. 15-day mortality and associated risk factors for hospitalized patients with COVID-19 in Wuhan, China: an ambispective observational cohort study. Intensive care medicine. 2020;46(7):1472-4.

37. World Health Organization. Report of the WHO-China Joint Mission on Coronavirus Disease 2019 (COVID-19) <https://www.who.int/docs/default-source/coronaviruse/who-china-joint-mission-on-covid-19-final-report.pdf>; 2020.

38. Xu PP, Tian RH, Luo S, Zu ZY, Fan B, Wang XM, et al. Risk factors for adverse clinical outcomes with COVID-19 in China: a multicenter, retrospective, observational study. Theranostics. 2020;10(14):6372-83.

39. Yang X, Yu Y, Xu J, Shu H, Xia J, Liu H, et al. Clinical course and outcomes of critically ill patients with SARS-CoV-2 pneumonia in Wuhan, China: a single-centered, retrospective, observational study. Lancet Respir Med. 2020;8(5):475-81.

40. Ye C, Zhang S, Zhang X, Cai H, Gu J, Lian J, et al. Impact of comorbidities on patients with COVID-19: A large retrospective study in Zhejiang, China. J Med Virol. 2020;16:16.

41. Yin Y, Zhou S, Zhang X, Li Z, Liu X, Jiang C, et al. Critically Ill Patients with COVID-19 in China: A Multicenter Retrospective Observational Study. [3/25/2020]. SSRN. 2020: <http://dx.doi.org/10.2139/ssrn.3562469>.

42. Yuan M, Yin W, Tao Z, Tan W, Hu Y. Association of radiologic findings with mortality of patients infected with 2019 novel coronavirus in Wuhan, China. PLoS ONE. 2020;15(3):e0230548.

43. Zeng H, Zhang T, He X, Du Y, Tong Y, Wang X, et al. Impact of Hypertension on Progression and Prognosis in Patients with COVID-19; A Retrospective Cohort Study in 1031 Hospitalized Cases in Wuhan, China. medRxiv. 2020:2020.06.14.20125997.

44. Zhang J, Yu M, Tong S, Liu LY, Tang LV. Predictive factors for disease progression in hospitalized patients with coronavirus disease 2019 in Wuhan, China. J Clin Virol. 2020;127:104392.

45. Zhang S, Guo M, Duan L, Wu F, Wang Z, Xu J, et al. Short Term Outcomes and Risk Factors for Mortality in Patients with COVID-19 in Wuhan, China: A Retrospective Study. [March 6, 2020]. SSRN. 2020: <http://dx.doi.org/10.2139/ssrn.3551390>.

46. Zhang S, Zhao J, Wu Z, Shang Y, Zheng J, Meng M, et al. Potential Predictors for Disease Progression and Medication Evaluation of 2019 Novel Coronavirus-Infected Pneumonia in Wuhan, China. [23/03/2020]. SSRN. 2020: <http://dx.doi.org/10.2139/ssrn.3551399>.

47. Zhou F, Yu T, Du R, Fan G, Liu Y, Liu Z, et al. Clinical course and risk factors for mortality of adult inpatients with COVID-19 in Wuhan, China: a retrospective cohort study. Lancet. 2020;395(10229):1054-62.

48. Zou X, Li S, Fang M, Hu M, Bian Y, Ling J, et al. Acute Physiology and Chronic Health Evaluation II Score as a Predictor of Hospital Mortality in Patients of Coronavirus Disease 2019. Critical care medicine. 2020;48(8):e657-e65.

49. Norooznezhad AH, Najafi F, Riahi P, Moradinazar M, Shakiba E, Mostafaei S. Primary Symptoms, Comorbidities, and Outcomes of 431 Hospitalized Patients with Confirmative RT-PCR Results for COVID-19. Am J Trop Med Hyg. 2020;24:24.

50. Gallo O, Locatello LG, Orlando P, Martelli F, Piccica M, Lagi F, et al. Cancer population may be paradoxically protected from severe manifestations of COVID-19. Journal of Infection. 2020;10:10.

51. Tomlins J, Hamilton F, Gunning S, Sheehy C, Moran E, MacGowan A. Clinical features of 95 sequential hospitalised patients with novel coronavirus 2019 disease (COVID-19), the first UK cohort. J Infect. 2020;81(2):e59-e61.

52. Mehta V, Goel S, Kabarriti R, Cole D, Goldfinger M, Acuna-Villaorduna A, et al. Case Fatality Rate of Cancer Patients with COVID-19 in a New York Hospital System. Cancer Discov. 2020;01:01.

53. Reilev M, Kristensen KB, Pottegård A, Lund LC, Hallas J, Ernst MT, et al. Characteristics and predictors of hospitalization and death in the first 9,519 cases with a positive RT-PCR test for SARS-CoV-2 in Denmark: A nationwide cohort. medRxiv. 2020:2020.05.24.20111823.

54. Mendy A, Apewokin S, Wells AA, Morrow AL. Factors Associated with Hospitalization and Disease Severity in a Racially and Ethnically Diverse Population of COVID-19 Patients. medRxiv. 2020;27:27.

55. Iftimie S, López-Azcona AF, Vicente-Miralles M, Descarrega-Reina R, Hernández-Aguilera A, Riu F, et al. Risk factors associated with mortality in hospitalized patients with SARS-CoV-2 infection. A prospective, longitudinal, unicenter study in Reus, Spain. bioRxiv. 2020:2020.05.29.122986.

56. Stroppa EM, Toscani I, Citterio C, Anselmi E, Zaffignani E, Codeluppi M, et al. Coronavirus disease-2019 in cancer patients. A report of the first 25 cancer patients in a western country (Italy). Fut Oncol. 2020;14:14.

57. Heili-Frades S, Minguez P, Fernández IM, Prieto-Rumeau T, González AH, de la Fuente L, et al. COVID-19 Outcomes in 4712 consecutively confirmed SARS-CoV2 cases in the city of Madrid. medRxiv. 2020:2020.05.22.20109850.

58. Pérez-Tanoira R, Pérez-García F, Romanyk J, Gómez-Herruz P, Arroyo T, González R, et al. Prevalence and risk factors for mortality related to COVID-19 in a severely affected area of Madrid, Spain. medRxiv. 2020:2020.05.25.20112912.

59. Peixoto VR, Vieira A, Aguiar P, Sousa P, Carvalho C, Thomas D, et al. COVID-19 :Determinants of Hospitalization, ICU and Death among 20,293 reported cases in Portugal. medRxiv. 2020:2020.05.29.20115824.

60. Zhao M, Wang M, Zhang J, Gu J, Zhang P, Xu Y, et al. Comparison of clinical characteristics and outcomes of patients with coronavirus disease 2019 at different ages. Aging (Albany NY). 2020;12(11):10070-86.

61. Rossi PG, Marino M, Formisano D, Venturelli F, Vicentini M, Grilli R. Characteristics and outcomes of a cohort of SARS-CoV-2 patients in the Province of Reggio Emilia, Italy. medRxiv. 2020:2020.04.13.20063545.

62. Lee H-Y, Ahn J, Kang CK, Won S-H, Park J-H, Kang CH, et al. Association of Angiotensin II Receptor Blockers and Angiotensin-Converting Enzyme Inhibitors on COVID-19-Related Outcome. [4/1/2020]. SSRN. 2020: <http://dx.doi.org/10.2139/ssrn.3569837>.

63. Ciceri F, Castagna A, Rovere-Querini P, De Cobelli F, Ruggeri A, Galli L, et al. Early predictors of clinical outcomes of COVID-19 outbreak in Milan, Italy. Clin Immunol. 2020;217:108509.

64. Wang Z, Zheutlin AB, Kao Y-H, Ayers KL, Gross SJ, Kovatch P, et al. Analysis of hospitalized COVID-19 patients in the Mount Sinai Health System using electronic medical records (EMR) reveals important prognostic factors for improved clinical outcomes. medRxiv. 2020:2020.04.28.20075788.
